# The Development of Therapeutic Antibodies That Neutralize Homologous and Heterologous Genotypes of Dengue Virus Type 1

**DOI:** 10.1371/journal.ppat.1000823

**Published:** 2010-04-01

**Authors:** Bimmi Shrestha, James D. Brien, Soila Sukupolvi-Petty, S. Kyle Austin, Melissa A. Edeling, Taekyung Kim, Katie M. O'Brien, Christopher A. Nelson, Syd Johnson, Daved H. Fremont, Michael S. Diamond

**Affiliations:** 1 Department of Medicine, Washington University School of Medicine, St. Louis, Missouri, United States of America; 2 Department of Pathology & Immunology, Washington University School of Medicine, St. Louis, Missouri, United States of America; 3 MacroGenics, Inc., Rockville, Maryland, United States of America; 4 Department of Biochemistry and Molecular Biophysics, Washington University School of Medicine, St. Louis, Missouri, United States of America; 5 The Midwest Regional Center of Excellence for Biodefense and Emerging Infectious Diseases Research, Washington University School of Medicine, St. Louis, Missouri, United States of America; 6 Department of Molecular Microbiology, Washington University School of Medicine, St. Louis, Missouri, United States of America; University of North Carolina, United States of America

## Abstract

Antibody protection against flaviviruses is associated with the development of neutralizing antibodies against the viral envelope (E) protein. Prior studies with West Nile virus (WNV) identified therapeutic mouse and human monoclonal antibodies (MAbs) that recognized epitopes on domain III (DIII) of the E protein. To identify an analogous panel of neutralizing antibodies against DENV type-1 (DENV-1), we immunized mice with a genotype 2 strain of DENV-1 virus and generated 79 new MAbs, 16 of which strongly inhibited infection by the homologous virus and localized to DIII. Surprisingly, only two MAbs, DENV1-E105 and DENV1-E106, retained strong binding and neutralizing activity against all five DENV-1 genotypes. In an immunocompromised mouse model of infection, DENV1-E105 and DENV1-E106 exhibited therapeutic activity even when administered as a single dose four days after inoculation with a heterologous genotype 4 strain of DENV-1. Using epitope mapping and X-ray crystallographic analyses, we localized the neutralizing determinants for the strongly inhibitory MAbs to distinct regions on DIII. Interestingly, sequence variation in DIII alone failed to explain disparities in neutralizing potential of MAbs among different genotypes. Overall, our experiments define a complex structural epitope on DIII of DENV-1 that can be recognized by protective antibodies with therapeutic potential.

## Author Summary

Dengue virus (DENV) is a mosquito-transmitted virus that infects 25 to 100 million humans annually and can progress to a life-threatening hemorrhagic fever and shock syndrome. Currently, no vaccines or specific therapies are available. Prior studies identified a highly neutralizing monoclonal antibody (MAb) against West Nile virus, a related flavivirus, as a candidate therapy for humans. In this study, we generated 79 new MAbs against the DENV type 1 (DENV-1) serotype, 16 of which strongly inhibited infection in cell culture. Using structural and molecular approaches, the binding sites of these inhibitory MAbs were localized to distinct regions on domain III of the DENV-1 envelope protein. We tested the protective capacity of all of the neutralizing MAbs in mice against infection by a strain of DENV-1 from a distinct genotype. Only two of the MAbs, DENV1-E105 and DENV1-E106, showed efficacy in a post-exposure treatment model, and these antibodies efficiently neutralized all five DENV-1 genotypes. Collectively, our studies define a complex structural binding site on domain III of the envelope protein for MAbs with therapeutic potential against DENV-1.

## Introduction

Dengue virus (DENV) is a member of the *Flaviviridae* family and is related to the viruses that cause yellow fever, and the Japanese, St. Louis, and the West Nile encephalitides [1]. DENV infection after mosquito inoculation causes a spectrum of clinical disease ranging from a self-limited febrile illness (DF) to a life threatening hemorrhagic and capillary leak syndrome (Dengue Hemorrhagic Fever (DHF)/Dengue Shock Syndrome (DSS)). Globally, there is significant diversity among DENV strains, including four distinct serotypes (DENV-1, DENV-2, DENV-3, and DENV-4) that differ at the amino acid level in the viral envelope proteins by 25 to 40 percent. There is additional complexity within a given DENV serotype, as genotypes vary further by up to ∼6% and 3% at the nucleotide and amino acid levels, respectively [2],[3]. At present, no approved antiviral treatment or vaccine is available, and therapy is supportive. DENV causes an estimated 25 to 100 million infections and 250,000 cases of DHF/DSS per year worldwide, with 2.5 billion people at risk [4],[5].

DENV is an enveloped virus with a single-stranded, positive-sense RNA genome [6]. The 10.7 kilobase genome is translated as a single polyprotein, which is cleaved into three structural proteins (C, prM/M, E) and seven nonstructural (NS) proteins (NS1, NS2A, NS2B, NS3, NS4A, NS4B, NS5). The mature DENV virion has a well-organized outer protein shell, a lipid membrane bilayer, and a less-defined inner nucleocapsid core [7],[8]. The ectodomains of DENV E proteins are assembled as dimers with each subunit comprised of three discrete domains [9]–[11]. Domain I (DI) is a central, eight-stranded β-barrel, which contains a single N-linked glycosylation site in most DENV strains. Domain II (DII) is a long, finger-like protrusion from DI and contains a second N-linked glycan that binds to DC-SIGN [12]–[15] and the highly conserved fusion peptide at its distal end. Domain III (DIII), which adopts an immunoglobulin-like fold, has been argued to contain a cell surface receptor recognition site [16]–[19]. Exposure to mildly acidic conditions in the trans-Golgi secretory pathway promotes virus maturation through a structural rearrangement of the flavivirus E proteins and cleavage of prM to M by a furin-like protease [20],[21]. Mature DENV virions are covered by 90 anti-parallel E protein homodimers, which are arranged flat along the surface with quasi-icosahedral symmetry.

Many flavivirus neutralizing antibodies recognize the structural E protein (reviewed in [22]). Serotype-specific MAbs against DENV reportedly have the greatest neutralizing activity [23],[24] although some sub-complex specific MAbs, which recognize some but not all DENV serotypes, also are inhibitory [25]–[27]. Protection in animals by antibodies correlates generally with the degree of neutralizing activity in vitro [24], [28]–[31]. Several type-specific strongly neutralizing antibodies against individual flaviviruses (e.g., West Nile virus (WNV), Japanese encephalitis virus (JEV), DENV-2, DENV-4, and yellow fever virus (YFV)) have been localized to epitopes in DI and DIII [26], [29], [32]–[42]. Based on their potency of neutralization and protection in vivo, anti-WNV humanized or human MAbs are in clinical development as candidate therapeutics (reviewed in [43]). Although type-specific neutralizing MAbs against DENV-1 have been characterized [44],[45], few have been mapped to specific amino acids or structural determinants. To date, only two neutralizing IgM against DENV-1 were mapped by neutralization escape selection to amino acids E279 and E293, which lie outside of DIII [46].

To develop neutralizing MAbs against DENV-1 with possible clinical potential, mice were infected with a genotype 2 strain of a DENV-1 virus, and boosted with recombinant protein of the homologous virus. 79 new MAbs against DENV-1 were generated: 15 of these were DIII-specific IgG and strongly neutralized the homologous DENV-1 strain. Of these, 9 were serotype-specific, 5 were sub-complex-specific, and only 1 MAb cross-reacted with all 4 serotypes of DENV. Several MAbs failed to neutralize at least one DENV-1 strain of a distinct genotype, suggesting that antibody recognition of the neutralizing epitopes on DIII varied among genotypes.

As a first step towards generating antibody therapeutics against DENV-1, we evaluated the protective capacity of these MAbs in an immunocompromised IFN-αβR^−/−^ × IFN-γR^−/−^ (AG129) mouse infection model with a heterologous DENV-1 genotype 4 strain. Among the strongly neutralizing antibodies tested, only four were highly protective. Two MAbs, DENV1-E105 and DENV1-E106, which neutralized all genotypes, were therapeutically active even when administered as a single dose four days after infection. These studies define the complexity of epitopes on DIII of DENV-1 that is recognized by highly protective antibodies with therapeutic potential.

## Results

### MAb generation

Previous studies with WNV and DENV-2 demonstrated that MAbs against DIII of the E protein protect against infection of cells [26],[27],[29],[32],[35],[41] and, in the case of WNV, in animals [29]. To date, few DENV-1 MAbs have been characterized functionally or mapped structurally. To develop neutralizing MAbs, mice were infected with the 16007 (genotype 2) prototype strain of DENV-1 and in some cases, boosted with recombinant DIII from the homologous DENV-1 strain. Initial immunization studies were performed with wild type BALB/c mice as done previously with WNV [29]. Because low neutralizing titers (<1/100) were achieved, likely because of poor replication of DENV-1, we switched to an immunodeficient (IFN-αβR^−/−^) strain in the C57BL/6 background. After screening more than 3,000 hybridoma clones as part of five independent fusions, we isolated and cloned 79 new MAbs that recognized cells infected with DENV-1 (**Table S1**).

All MAbs were initially tested semi-quantitatively for neutralization of the homologous DENV-1 strain by a single endpoint plaque reduction assay in Vero or BHK21-15 cells using neat hybridoma supernatant (∼10 µg/ml). Of the MAbs generated, 18 showed no inhibitory activity (<15% neutralization), 45 had modest inhibitory activity (15–75% neutralization) and 16 were strongly inhibitory (>95% neutralizing). All MAbs were screened for E protein domain recognition using yeast that expressed DENV-1 DI-II or DIII on their surface (**Table S1**): 14 bound to yeast expressing DI-DII, 53 recognized DIII, and the remaining 12 did not bind either DI-DII or DIII, indicating they recognized epitopes that are not present on the truncated yeast-expressed forms of DENV-1 E protein. All MAbs were tested for cross-reactivity using cells infected with different serotypes of DENV or WNV. Five of the fourteen DI-DII-specific MAbs and 26 of the 53 DIII-specific MAbs cross-reacted with other serotypes of DENV. Only 3 of the 78 MAbs bound all DENV serotypes and WNV. One of the neutralizing MAbs, DENV1-E50, was of the IgM isotype and not evaluated further here.

### Characterization of strongly neutralizing MAbs against DENV-1

Studies with neutralizing mouse MAbs against DENV-2 defined a type-specific epitope on the lateral ridge of DIII (DIII-LR) and an adjacent more conserved β-strand termed the sub-complex-specific DIII A-strand epitope [25]–[27],[41]. As our single endpoint titer neutralization experiments showed that all 15 inhibitory IgG MAbs localized to DIII of DENV-1, we analyzed their reactivity patterns with other strains. We found that 9 of the 15 neutralizing IgG MAbs were serotype-specific and showed no cross-reactivity to DENV-2, DENV-3, DENV-4 or WNV-infected cells. Five MAbs were sub-complex specific and bound some but not all DENV serotypes. Only one MAb, DENV1-E102, cross-reacted with all DENV serotypes but not with WNV-infected cells (**Table 1**).

**Table 1:** Binding of MAbs to cells infected with other DENV serotypes.

MAb	Neutralization	Specificity	Binding to virus-infected cells^a^			
			DENV-1	DENV-2	DENV-3	DENV-4
			16007	16681	16652	H241
DENV1-E90	Strong	Sub-complex^b^	+++	−	−	+++
DENV1-E95	Strong	Type	+++	−	−	−
DENV1-E98	Strong	Sub-complex	+++	+	+++	−
DENV1-E99	Strong	Sub-complex	+++	+++	−	−
DENV1-E100	Strong	Type	+++	−	−	−
DENV1-E101	Strong	Type	+++	−	−	−
DENV1-E102	Strong	Cross-reactive	+++	+++	+	+++
DENV1-E103	Strong	Type	+++	−	−	−
DENV1-E104	Moderate	Type	+++	−	−	−
DENV1-E105	Strong	Type	+++	−	−	−
DENV1-E106	Strong	Sub-complex	+++	−	−	+++
DENV1-E108	Strong	Type	+++	−	−	−
DENV1-E111	Strong	Type	+++	−	−	−
DENV1-E112	Strong	Type	+++	−	−	−
DENV1-E113	Strong	Sub-complex	+++	+++	−	+++

Raji-DC-SIGN-R cells were infected with the indicated DENV serotypes and relative binding was determined by flow cytometry based on the mean fluorescence intensity after staining with MAbs.

a +++, strong binding (40–100%) to infected cells; +, weak binding (15–40%) to infected cells; −, no appreciable binding detected.

b sub-complex MAbs recognize some but not all DENV serotypes.

### Genotype-specific reactivity of anti-DENV-1 MAbs

As variation exists in the genetic composition of viruses within a given DENV serotype, an intra-serotype genotype classification system was developed [2],[3], with a given genotype of a serotype having no more than 6% nucleotide sequence divergence in the E gene [47]. To address whether the neutralizing MAbs were affected by the variation within the serotype, we tested reactivity and neutralizing potential against individual strains that encompass the five distinct DENV-1 genotypes: Asian genotype 1 (strain TVP-2130), Thai genotype 2 (strain 16007), Malaysian genotype 3 (TVP-5175), South Pacific genotype 4 (strain West-Pac 74), and American/African genotype 5 (strain 3146 SL).

#### (a) Binding to different DENV-1 genotypes

**Figure 1:**
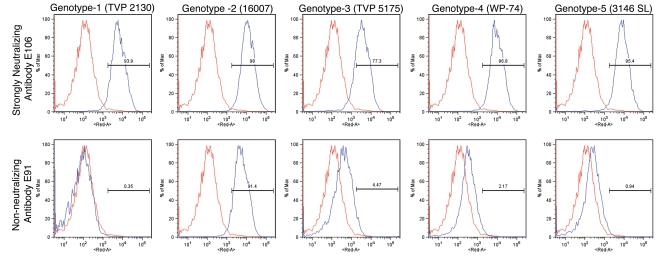
Binding of MAbs to cells infected with different genotypes of DENV-1. C6/36 insect cells were infected with strains of DENV-1 virus corresponding to all five genotypes. After fixation and permeabilization, MAbs were incubated with infected cells and binding was assessed by flow cytometry. The data shown are representative histograms from two independent experiments with a neutralizing (DENV1-E106, *upper panels*) and non-neutralizing (DENV1-E91, *lower panel*s) MAb.

To determine whether antibodies recognized different genotypes of DENV-1, we infected Raji-DC-SIGN-R or C6/36 cells (depending on permissiveness) with strains of the five different genotypes of DENV-1. Infected cells were incubated with hybridoma supernatants and analyzed by flow cytometry. All neutralizing MAbs tested efficiently recognized genotypes 1, 2, 4, and 5. However, a few of the MAbs (DENV1-E100, DENV1-E104, and DENV1-E111) showed reduced binding to cells infected with the genotype 3 strain (**Table 2**). In contrast, other DIII-specific non-neutralizing MAbs (e.g., DENV1-E91) showed reduced binding to several of the heterologous DENV-1 genotypes (**Fig 1**).

**Table 2:** MAb binding to cells infected with different genotypes of DENV-1 virus.

MAb	Binding to virus^a^				
	Genotype 1	Genotype 2	Genotype 3	Genotype 4	Genotype 5
	TVP-2130	16007	TVP-5175	West Pac-74	3146-SL
DENV1-E90	93	98	83	95	95
DENV1-E95	94	98	81	95	95
DENV1-E98	97	98	83	96	97
DENV1-E99	96	98	84	96	97
DENV1-E100	93	98	***70***	94	95
DENV1-E101	93	98	82	95	96
DENV1-E102	92	98	78	94	94
DENV1-E103	94	98	83	95	95
DENV1-E104	93	98	***71***	94	92
DENV1-E105	89	98	75	94	95
DENV1-E106	94	98	75	95	95
DENV1-E108	93	98	77	95	94
DENV1-E111	90	98	***57***	93	92
DENV1-E112	94	98	81	95	95
DENV1-E113	90	98	74	93	93

a Depending on the strain, Raji-DC-SIGN-R or C6/36 cells were infected with different genotypes of DENV-1 viruses. Fixed and permeabilized infected cells were incubated with the indicated MAbs (10 µg/ml) to determine the binding efficiency by flow cytometry. The data shown are the mean percentage of positive cells and derived from three independent experiments. Values underlined and italicized are statistically different (P<0.05).

#### (b) Neutralizing potential of MAbs against different DENV-1 genotypes

Although most MAbs retained binding to cells infected with many of the genotypes, we questioned whether this translated into efficient cross-genotypic neutralization. A recent study of neutralization escape mutants with human MAbs against WNV showed that retention of binding did not necessarily sustain neutralizing activity [48]. To evaluate this, we assessed semi-quantitatively the inhibitory activity of hybridoma supernatants of 15 neutralizing MAbs against all five genotypes by a standard plaque reduction assay with 10^2^ PFU of virus in Vero or BHK21-15 cells, depending on the strain (**Table 3**). Only 8 of 15 hybridoma supernatants neutralized strains from all five genotypes by greater than 90%, with three MAbs (DENV1-E103, DENV1-E105, and DENV1-E106) showing 100% neutralization of all genotypes. Several MAbs showed reduced neutralizing potential for individual genotypes even at the relatively high (∼10 µg/ml) concentration of MAb used in the assay. For example, 9, 12, 12, and 14 MAbs neutralized infection by DENV genotypes 1, 3, 4, and 5, respectively. This was especially surprising for the genotype 1 strain as no appreciable reduction in binding to infected cells was observed (**Table 2**). As an example, DENV1-E104 showed relatively normal binding to the genotype 1 strain yet showed a 92% loss in neutralization activity. Some MAbs (DENV1-E100 and DENV1-E111), which had decreased binding to genotype 3 infected cells, also showed reduced inhibitory activity. Others (e.g., DENV1-E104) sustained normal inhibitory activity despite attenuated binding to fixed, permeabilized cells. Although more studies are necessary, this could reflect an epitope that is more sensitive to fixation in the context of recognition of specific genotypes.

**Table 3:** MAb neutralization of different DENV-1 genotypes.

MAb	aPercent Neutralization (%)				
	Genotype 1	Genotype 2	Genotype 3	Genotype 4	Genotype 5
	TVP-2130	16007	TVP-5175	West Pac-74	3146-SL
DENV1-E90	95	100	93	97	100
DENV1-E95	93	100	94	***72***	93
DENV1-E98	90	100	100	99	100
DENV1-E99	95	100	97	96	100
DENV1-E100	100	100	96	97	99
DENV1-E101	98	100	100	92	100
DENV1-E102	***79***	100	100	98	100
DENV1-E103	100	100	100	100	100
DENV1-E104	***8***	96	***51***	***55***	***48***
DENV1-E105	100	100	100	100	100
DENV1-E106	100	100	100	100	100
DENV1-E108	***69***	100	86	97	95
DENV1-E111	***15***	100	***63***	85	91
DENV1-E112	***46***	100	***76***	***79***	94
DENV1-E113	***71***	100	97	92	100

a Neutralizing activity was determined semi-quantitatively by single endpoint plaque reduction assay on BHK21 or Vero cells with neat hybridoma supernatant cells and 10^2^ PFU of the indicated DENV-1 genotype. The data was derived from at least two independent assays performed in duplicate. Highlighted, italicized values indicate a genotypic specific reduction in neutralizing potential of a given MAb using neat hybridoma supernatant of greater than 20%.

To more rigorously characterize the neutralizing potency, we purified MAbs and assessed their inhibitory activity against the homologous genotype 2 (16007) strain in cell culture by performing a dose-response curve and determining the concentration of MAb (PRNT50, expressed here as ng/ml of antibody) that blocked plaque formation by 50% (**Table 4**). For the homologous genotype 2 16007 strain, 8 of 15 MAbs potently neutralized infection with PRNT50 values below 5 ng/ml. This value is significant as it is lower than that observed with our most inhibitory anti-WNV MAb (E16), which functions successfully as a post-exposure therapeutic agent [29],[49],[50]. Six other MAbs also showed strong inhibitory activity with PRNT50 values against 16007 of between 10 and 70 ng/ml. Only one MAb, E104 showed modest activity with a PRNT50 value of ∼600 ng/ml.

**Table 4:** PRNT50 values of MAbs against a homologous and heterologous DENV-1 strain.

MAb	Genotype 2	Genotype 4	Fold reduction (WP 74/16007)
	16007	West Pac-74	
	PRNT50 (ng/ml) ± SD	PRNT50 (ng/ml) ± SD	
DENV1-E90	1.9±1.9	1161±241	611
DENV1-E95	14±3.1	8561±4480	612
DENV1-E98	13±2.5	757±212	58
DENV1-E99	11±3.9	803±116	73
DENV1-E100	24±5.0	145±42	6
DENV1-E101	0.8±0.5	1029±327	1286
DENV1-E102	1.1±0.4	575±546	522
DENV1-E103	0.7±0.3	542±192	774
DENV1-E104	590±105	23068±2720	39
DENV1-E105	0.5±0.09	20±9.7	40
DENV1-E106	0.6±0.3	13±7.5	21
DENV1-E108	70±9.1	3912±1930	56
DENV1-E111	3.7±1.6	15198±4905	4107
DENV1-E112	42±13	18891±3331	450
DENV1-E113	1.3±0.3	12498±8911	9614

Neutralizing activity was determined by plaque reduction assay on BHK21 with increasing concentrations of purified MAb cells and 10^2^ PFU of the indicated DENV-1 genotype. The data was derived from three independent experiments performed in duplicate. PRNT50 values were calculated by non-linear regression analysis and SD indicates the standard deviations. The column on the right was obtained by dividing the PRNT50 values from West Pac-74 by those of 16007 for a given MAb.

Given our findings of genotype variation with respect to single endpoint neutralization using hybridoma supernatants, we repeated the dose-response curve analysis with a heterologous genotype 4 (West Pac-74) (**Table 4**). Surprisingly, the neutralizing activity was markedly decreased for all MAbs against the heterologous DENV-1 genotype (e.g., 6 to 9600-fold for DENV1-E100 and DENV1-E113, respectively). Only 2 MAbs, DENV1-E105 and DENV1-E106, retained strong neutralizing activity (≤20 ng/ml) against the West Pac-74 strain. Seven of 15 MAbs neutralized West-Pac 74 poorly with PRNT50 values greater than 1 µg/ml of antibody. Thus, the neutralizing efficiency of several DIII-specific anti-DENV-1 MAbs was significantly altered against strains of a different DENV-1 genotype.

### Crystal structure of DENV-1 DIII and analysis of genotypic variation

**Figure 2:**
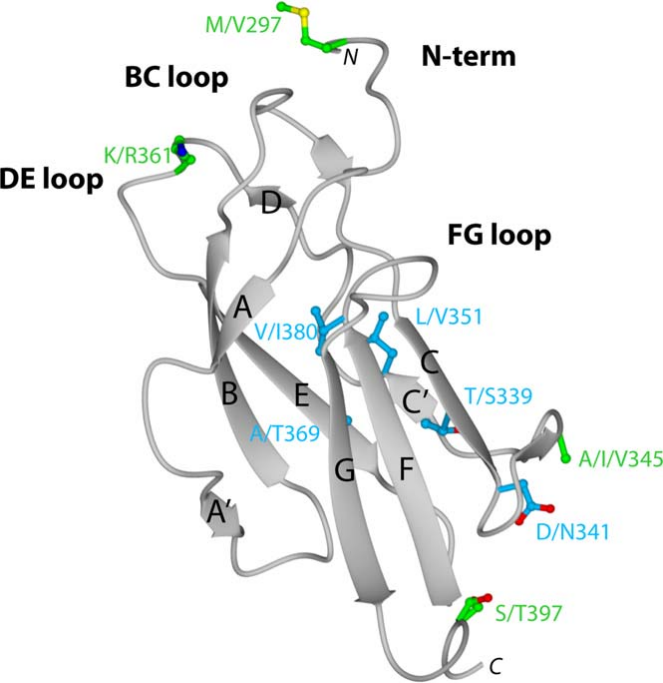
Crystal structure of DENV-1 DIII (strain 16007) and analysis of genotypic variation. Ribbon diagram of Ig-like fold of DENV-1 DIII with β-strands and loops labeled accordingly after solution of the X-ray crystallographic structure at 2.25 Å resolution. Amino acid sequences in DIII that vary among the five different DENV-1 genotypes are indicated, with solvent inaccessible and accessible residues depicted in blue and green, respectively.

**Figure 3:**
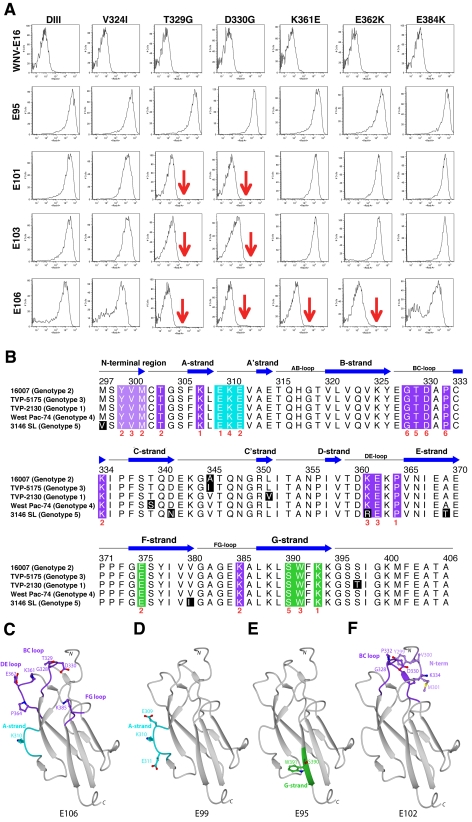
Epitope localization of anti-DENV-1 MAbs. **A**. Flow cytometry histograms of loss-of-function DIII variants (V324I, T329G, D330G, K361E, E362K, and E384K) with individual neutralizing MAbs. Representative histograms are shown for the MAbs WNV E16 (negative control), DENV1-E95, DENV1-101, DENV1-103, and DENV1-106 with the wild type DIII and each of the variants. Data shown are representative of three independent experiments. **B**. Sequence alignment of different DENV-1 genotypes and mapping of neutralizing MAbs. The sequence and secondary structure of DIII from DENV-1 (strain 16007, genotype 2) E protein is aligned with other DENV-1 genotypes (genotype 1, strain TVP-2130; genotype 3, strain TVP 5175; genotype 4, strain Western Pacific-74; genotype 5, strain 3146 Sri Lanka). The secondary structure of DENV-1 E DIII residues 294 to 395 from the strains that had not been crystallized was predicted by DSSP [112] using the 16007 strain coordinates. Black blocks highlight residues of genotypic variation. The results of the yeast surface-display epitope mapping (see **Table 5**) are denoted underneath in red to indicate the number of neutralizing MAbs in our panel that lose binding when a specific amino acid is changed. Colored boxes correspond to specific neutralizing antibody and structural recognition determinants: N-terminal region, light purple; lateral ridge (BC, DE, and FG loops), dark purple; A-strand, cyan; and F- and G-strand, green. **C–F**. Localization of neutralizing epitopes on DENV-1 DIII as determined by yeast surface display. Structure of DENV-1 DIII (strain 16007) with amino acid residues marked in blue that significantly affect binding of neutralizing MAbs (**C**) DENV1-E106, (**D**) DENV1-E99, (**E**) DENV1-E95, and (**F**) DENV1-E102.

To begin to understand the disparity in inhibitory potential of the DIII-specific neutralizing MAbs among different genotypes, we determined the X-ray crystal structure of recombinant DIII of the 16007 (genotype 2) strain at 2.25-Å resolution (**Fig 2** **and Table S2**). Among the DENV-1 strains that represent the five different genotypes used in this study, variation in DIII was limited to nine residues, six of which were conservative substitutions (**Fig 2** **and** **Fig 3B**). Four of the conservative substitutions (V/I380, L/V351, T/S339, and D/N341) are predicted to be partially or completely solvent inaccessible in the isolated DENV-1 DIII crystal structure and cryoelectron microscopy reconstruction of the mature virion [51], and thus, should not directly affect antibody engagement. In contrast, the conservative substitution K/R361 should be accessible due to its location in the DE loop. S/T397, the remaining conservative substitution is located after the G-strand in a region that links DIII with the first α-helix of the stem anchor motif. Of the three non-conservative substitutions, A/T369 is completely buried and although A/I/V345 is largely solvent accessible in the DIII crystal structure, it is likely buried in the virion according to cryoelectron microscopy reconstructions. The remaining non-conservative substitution (M/V297) occurs in the N-terminal linker region that connects to DI, which is solvent accessible in the DIII crystal structure, but less so in the mature virion structure. In summary, amino acid variation in DIII among the five different DENV-1 genotypes is modest, with the majority of changes being solvent inaccessible. Indeed, none of the observed genotypic variation occurs in residues previously implicated as critical to the creation of neutralizing antibody epitopes in DENV or related flaviviruses.

### Epitope mapping of DIII neutralizing MAbs

On the basis of studies that mapped DIII-specific MAbs against WNV and DENV-2 [25]–[27],[29],[41], we initially used reverse genetics to engineer corresponding mutations (S305P, K307E, E309K, K310E, V324I, T329G, D330G, K361E, E362K, E384K, K385E, and K393E) on residues of the BC, DE, and FG loops and the A β-strand (A-strand) of DIII of the genotype 2 (16007) strain, and displayed these variants on the surface of yeast. All DIII-specific MAbs were screened for loss-of-binding to the mutants to identify potentially critical recognition residues. Using this strategy, we localized binding of seven MAbs (DENV1-E100, DENV1-E101, DENV1-E102, DENV1-E103, DENV1-E104, DENV1-E105, and DENV1-E106) to individual or combinations of amino acids on the BC, DE, and FG loops of the lateral ridge of DIII (**Table 5** **,** **Fig 3A, B, and C**). Analogously, five MAbs (DENV1-E99, DENV1-E100, DENV1-E104, DENV1-E106, and DENV1-E113) exhibited reduced binding after mutation of A-strand (K307, E309, K310, and/or E311) residues (**Fig 3D**). Three of the neutralizing MAbs (DENV1-E95, DENV1-E111, and DENV1-E112), however, showed no appreciable reduction in binding with analogous mutations in the lateral ridge or A-strand residues that were identified from DENV-2 yeast screens.

**Table 5:** Summary of MAb binding to DENV-1 DIII mutants expressed on the surface of yeast.

MAb	Y299N	V300M	V300E	M301I	T303I	S305P	K307E	E309K	K310E	E311K	V324I	G328E	T329G	D330G	P332S	K334N	Q340H	K343I	P356S	K361E	E362K	P364A	E370D	E375V	E384K	K385E	S390I	S390N	S390R	W391L	K393E	A404S
**E90**	80	100	**2**	97	28	95	44	40	83	90	55	**4**	100	72	**10**	86	93	97	84	100	100	94	100	78	100	100	88	90	100	100	100	84
**E95**	95	100	100	95	86	73	58	71	91	72	51	100	100	99	89	89	98	93	100	100	99	81	100	73	100	100	**<1**	**<1**	**<1**	**<1**	100	91
**E98**	91	94	100	47	66	77	50	75	23	94	53	100	100	94	80	85	92	79	100	100	100	75	99	**<1**	100	99	**<1**	41	**<1**	68	100	79
**E99**	82	98	100	100	68	78	23	**15**	**5**	**7**	52	100	100	100	90	83	82	78	99	100	99	73	100	100	100	99	78	76	100	100	100	78
**E100**	100	100	37	31	100	73	**1**	93	33	100	28	100	**<1**	**2**	42	100	100	100	82	**<1**	**<1**	100	48	100	90	53	100	100	80	100	39	100
**E101**	100	100	61	63	**5**	82	74	55	92	88	50	**6**	**<1**	**8**	**6**	100	76	94	100	98	100	87	100	100	100	100	100	91	95	99	100	62
**E102**	**<1**	**1**	**<1**	**2**	86	71	72	74	67	81	49	**9**	99	**15**	**2**	**5**	71	100	67	100	100	88	95	70	100	100	69	73	61	81	100	71
**E103**	50	74	100	73	**9**	72	51	71	76	76	100	**<1**	**<1**	**2**	**1**	67	71	92	82	99	100	88	97	75	100	100	62	58	75	100	100	68
**E104**	86	91	73	58	99	100	100	73	**15**	**4**	100	49	100	26	38	89	65	100	100	45	48	100	98	**<1**	83	**10**	**<1**	**<1**	**<1**	**<1**	**16**	69
**E105**	100	100	80	51	100	100	24	100	23	71	48	100	**1**	23	**2**	66	40	45	70	**1**	**15**	43	76	100	100	96	57	46	100	100	92	69
**E106**	83	79	85	57	65	76	75	100	**16**	100	100	**2**	**<1**	**1**	22	74	42	67	100	**3**	**3**	**5**	67	100	37	**12**	56	60	91	100	93	58
**E108**	100	100	100	100	100	100	99	100	92	36	98	100	62	100	100	100	100	100	54	91	99	100	37	100	100	96	**5**	33	**5**	62	97	100
**E111**	77	100	100	98	67	100	100	49	62	96	100	100	100	100	80	81	84	76	100	100	100	72	100	100	100	100	72	72	100	100	100	74
**E112**	78	100	100	100	66	100	94	100	68	98	100	100	100	99	63	73	80	76	100	100	100	67	100	100	100	99	41	**8**	78	**1**	100	73
**E113**	**1**	**1**	**<1**	**2**	65	100	100	63	**7**	71	52	**4**	89	75	**4**	**13**	100	89	22	99	65	91	100	100	73	92	85	90	100	100	52	96

Values shown were achieved by dividing the total fluorescence product (percent positive population×mean linear fluorescence intensity) of a mutant for each individual antibody by the total fluorescence product of the wild type DIII×100. Values in bold indicate reductions in mAb binding of greater than or equal to 80% for a given mutation. Underlined values show a reduction between 50 and 79%. The results are the average of between three and five independent experiments for each mutant and each antibody.

Forward genetic screens subsequently were performed to define additional residues that affected MAb binding. Error-prone PCR introduced random point mutations within DIII of DENV-1 E protein. Yeast were incubated sequentially with an Alexa Fluor 647-conjugated individual MAb and an Alexa Fluor 488-conjugated oligoclonal pool of MAbs to eliminate mutants that abolished surface expression of DIII (see Materials and Methods). Yeast that selectively lost expression of individual MAb epitopes were identified, subjected to plasmid recovery, sequenced, and tested for reactivity by flow cytometry against the remainder of the MAb panel (**Table 5**). In total, 21 DIII residues were identified that when changed resulted in a decrease in yeast staining by one or more MAbs, with mutations in BC-loop residues most frequently observed (**Fig 3B**). Only one of these residues (K/R361) is variable amongst the five genotypes, an unexpected observation based on the distinct genotypic specificities of the different MAbs.

The two most protective MAbs in vivo (see below), DENV1-E105 (type-specific) and DENV1-E106 (sub-complex specific), lost binding when residues on the BC (G328, T329 and D330), DE (K361E and E362K), and FG (K385) loops of the DIII-LR region were altered. Similarly, other protective MAbs (DENV1-E100 and DENV1-E103) also lost binding when residues in the BC or DE loop were altered. Type-specific MAbs (DENV1-E95, DENV1-E101, DENV1-E104, DENV1-E108, DENV1-E111, and DENV1-E112) that inhibited genotype 2 (16007) yet poorly neutralized the genotype 4 (West Pac-74) strain mapped to additional sites in DIII. DENV1-E95, DENV1-E104, DENV1-E108, and DENV1-E112 exhibited reduced binding with mutations in the G-strand (S390, W391, and K393) (**Fig 3E**) whereas DENV1-E101 was affected by changes in the N-terminal linker (T303) and BC-loop (G328, T329, and D330). Surprisingly, none of the mutations altered DENV-E111 binding, and forward genetic screens with this MAb also failed to identify a loss-of-binding variant (data not shown). DENV1-E104, which showed the weakest neutralizing activity, localized to residues in the A (K310 and E311), F (E375), and G (S390 and W391) β-strands.

Because of analogous studies with DENV-2, we anticipated that sub-complex-specific and cross-reactive MAbs would recognize the A-strand epitope [25]–[27]. Indeed, mutation of S305, K307, E309, K310, and E311 reduced binding of several sub-complex MAbs (DENV1-E98, DENV1-E99, DENV1-E106, and DENV1-E113) in our panel. For the cross-reactive neutralizing MAb DENV1-E102, its epitope localized to residues in the N-terminal linker region (Y299, V300, and M301) and the BC loop (D330 and P332) (**Fig 3F**). Although Y299, M301, and P332 are highly conserved among strains of different DENV serotypes, V300 is specific for DENV-1 and D330 is present in DENV-1 and DENV-3; this may explain why DENV1-E102 fails to neutralize DENV-2 and DENV-3 efficiently (S. Sukupolvi-Petty, J. Brien, and M. Diamond, unpublished results).

Despite the extensive epitope mapping data (summarized in **Fig 3B** **and** **4**), no structural explanation was readily apparent for the decrease in neutralization of the genotype 4 West Pac-74 strain by several antibodies. MAbs that showed markedly depressed neutralization of West Pac-74 localized to residues that were instead conserved among DENV-1 genotypes. To begin to address this, we evaluated whether individual MAbs differentially recognized DIII from 16007 or West Pac-74 strains when expressed on yeast; the DIII of these two strain differ by only two amino acids at positions 339 and 345 in the C strand and C-C′ loop (**Fig 3B**). Notably, all neutralizing MAbs equivalently recognized yeast displaying DIII of 16007 or West Pac-74 over a range of concentrations (**Fig 5A**, and data not shown). Thus, sequences differences in DIII of West Pac-74 and 16007 did not explain the differential patterns of neutralization. Because of this, we hypothesized that sequences variation in other domains of the West Pac-74 E protein could directly alter MAb binding or perhaps influence the display of DIII on the infectious virion. To test this, we performed a virion capture ELISA with 16007 and West Pac-74 viruses and selected MAbs having different neutralization profiles against the two strains (**Fig 5B**). DENV1-E98, which exhibited a ∼50-fold reduction in neutralization showed little difference in binding over several concentrations of antibody. In contrast, DENV1-E111, which had ∼4,000-fold less neutralizing activity, exhibited a depressed ability to bind West Pac-74 compared to 16007. Surprisingly, DENV1-E90 and DENV1-E113, which had ∼600 to 9,600-fold differences in PRNT50 values with West Pac-74 compared to 16007 showed virtually no variation in binding in the capture ELISA. Thus, differential neutralization of genotypes 2 and 4 of DENV-1 could be explained by disparate binding to the virion for only a subset of MAbs. For MAbs like DENV1-E90 and DENV1-E113, the profile was similar to that observed with escape variants of a human anti-WNV MAb; in that case, mutations that abolished neutralizing activity did not appear to affect antibody binding to virus [48].

**Figure 4:**
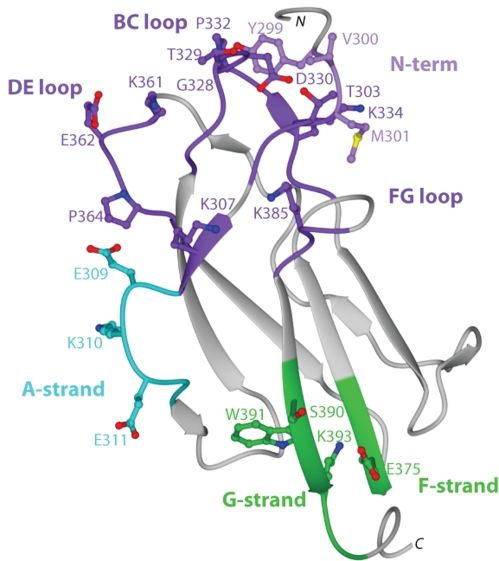
Summary of neutralizing antibody and structural recognition determinants on DENV-1 DIII. Data from yeast surface display (**Table 5**) and sequence alignment (**Fig 3B**) were mapped onto the crystal structure of DENV-1 DIII to visualize the composite epitopes of different neutralizing MAbs. Recognition sites include N-terminal region, light purple; lateral ridge (BC, DE, and FG loops), dark purple; A-strand, cyan; and F- and G-strand, green.

**Figure 5:**
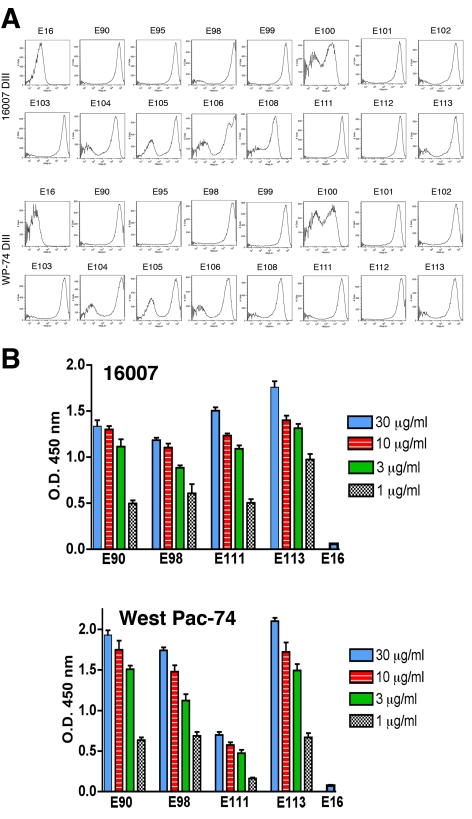
Binding of MAbs to DIII or virions of 16007 and West Pac-74. **A**. MAb binding to DIII of 16007 or West Pac-74 on yeast. Flow cytometry histograms of DIII of 16007 or West Pac-74 (different at residues 339 and 345) with neutralizing MAbs. Data shown are representative of two independent experiments. **B**. A capture ELISA was used to detect binding of MAbs to 16007 and West Pac-74 virions. Microtiter plates were coated with the indicated MAbs, incubated with 2×10^5^ PFU of 16007 or West Pac-74 at room temperature, and with biotinylated anti-E MAbs. The data is an average of several separate experiments performed in duplicate, and error bars indicate standard deviation.

### In vivo protection studies with neutralizing MAbs

Pre-exposure passive transfer of neutralizing MAbs against WNV, DENV-2, and DENV-4 protects against lethal infection in wild type and immunodeficient mice [24], [29], [52]–[56]. To confirm that neutralizing anti-DENV-1 MAbs protect in vivo and begin to explore the possibility for antibody therapy, we evaluated the efficacy of our panel of inhibitory MAbs against West Pac-74, the heterologous genotype 4 DENV-1 strain. The heterologous strain was selected for two reasons: (i) the West Pac-74 strain was the only DENV-1 isolate in our collection that caused 100% lethality in an intraperitoneal challenge model in AG129 mice; and (ii) a possible antibody therapeutic must demonstrate efficacy against DENV-1 strains of different genotypes.

A single dose (500 µg) of neutralizing MAbs against DENV-1 was administered as prophylaxis one day prior to infection with 10^6^ PFU of West Pac-74. Two MAbs (DENV1-E105 and DENV1-E106) protected 100% of mice compared to PBS or irrelevant MAb controls (P<0.0001), which had a 0% survival rate (**Table 6**). Three other MAbs (DENV1-E99, DENV1-E100, and DENV1-E103) exhibited strong yet incomplete protection with 60 to 80% survival rates. However, nine neutralizing MAbs (DENV1-E90, DENV1-E95, DENV1-E98, DENV1-E101, DENV1-E102, DENV1-E108, DENV1-E111, DENV1-E112 and DENV1-E113) protected inefficiently, with an 11 to 38% survival rate. Although the survival rate was relatively low for these MAbs, the mean time to death (MTD) was protracted (23±6.1 to 40±8.6 days) compared to untreated, infected mice (20±3.3 days). One neutralizing MAb, DENV1-E104 showed no protection against lethality although a longer MTD was observed. In general, the protective efficacy in vivo correlated with neutralizing activity against the West Pac-74 genotype 4 strain. As an example, DENV1-E105 and DENV1-E106 had low PRNT50 values (13 to 20 ng/ml) for West Pac-74 and protected against infection whereas DENV1-E104 and DENV1-E112, which protected poorly, had PRNT50 values (18 to 23 µg/ml) that were almost 1,000-fold different.

**Table 6:** In vivo protection of AG129 mice from West Pac-74 (genotype 4) after prophylaxis with neutralizing anti-DENV-1 MAbs.

MAb	Dose (µg/mouse)	Survival	MTD^a^ ± SD	P value^b^
No MAb	0	0/12	20±3	
WNV E16	500	0/5	18±2	NS
DENV1-E90	500	2/9	37±7	<0.0001
DENV1-E95	500	2/10	36±11	<0.0001
DENV1-E98	500	2/8	34±14	0.002
DENV1-E99	500	6/8	52±3	<0.0001
DENV1-E100	500	6/10	35±17	<0.0001
DENV1-E101	500	3/8	34±12	0.0005
DENV1-E102	500	1/9	23±6	0.03
DENV1-E103	500	7/10	29±14	<0.0001
DENV1-E104	500	0/8	26±8	0.03
DENV1-E105	500	8/8	NA	<0.0001
DENV1-E106	500	10/10	NA	<0.0001
DENV1-E108	500	2/8	40±9	<0.0001
DENV1-E111	500	2/8	28±8	0.001
DENV1-E112	500	1/8	25±11	0.03
DENV1-E113	500	1/8	34±11	0.001

Mice were administered with 500 µg of indicated MAbs one day before infection with 10^6^ PFU of West Pac-74 by an IP route. Mice were monitored for survival for 60 days after infection. NS, not statistically different from PBS control.

a Mean time to death (MTD) ± standard deviations (SD) refers to the mean time to death of the animals that succumbed to fatal infection. NA, indicates that no animals died in the presence of this MAb, and thus, the value was not calculated.

b P values were calculated using the log rank test of the Kaplan Meier survival curve by comparing the no antibody treated and antibody treated mice.

**Figure 6:**
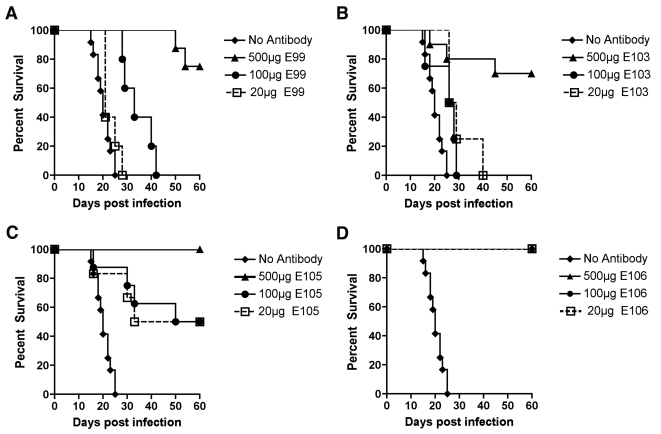
Dose response of protective efficacy MAbs in mice infected with DENV-1. AG129 mice were passively transferred saline or 20, 100, or 500 µg of (**A**) DENV1-E99, (**B**) DENV1-E103, (**C**) DENV1-E105, and (**D**) DENV1-E106 MAbs one day before infection with 10^6^ PFU of West Pac-74 (genotype 4) by an IP route. Mice were monitored for survival for 60 days after infection. The survival curves were constructed from data of two independent experiments. The number of animals for each antibody dose ranged from 5 to 10 per group.

To begin to define the therapeutic potential of the highly protective MAbs (DENV1-E99, DENV1-E103, DENV1-E105, and DENV1-E106), we administered 5 and 25-fold lower doses (100 µg and 20 µg) one day prior to IP infection of AG129 mice with 10^6^ PFU of West Pac-74. The lower doses of DENV1-E99 and DENV1-E103 provided sub-optimal protection against lethal infection (**Fig 6A and B**), with only a delay in the MTD observed. In comparison, ∼50% of mice survived West Pac-74 infection after receiving a single dose of 20 or 100 µg of DENV1-E105 antibody (**Fig 6C**, P≤0.003). More strikingly, a single 20 or 100 µg injection of DENV1-E106 completely protected DENV-1 infected mice (**Fig. 6D**, P<0.0001). These data suggest that DENV1-E106 was the most protective MAb in our panel of neutralizing antibodies against infection by the heterologous strain, DENV-1 West Pac-74.

### Therapeutic studies with neutralizing MAbs

**Figure 7:**
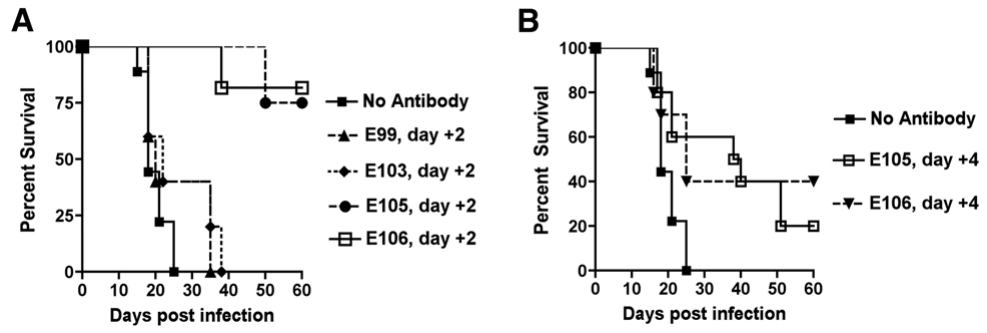
Therapeutic efficacy of strongly neutralizing antibodies in mice after DENV-1 infection. Mice were administered saline or a single 500 µg dose of DENV1-E105 or DENV1-E106 MAbs at day (**A**) 2 or (**B**) 4 after infection with 10^6^ PFU of West Pac-74 (genotype 4) by an IP route. Mice were monitored for survival for 60 days after infection. The number of animals for each antibody ranged from 10 to 11 per group.

Antibody-based therapeutics against DENV will need to be administered after infection. To assess the therapeutic efficacy of strongly neutralizing anti-DENV-1 MAbs, mice were infected with 10^6^ PFU of West Pac-74 and two days after infection, a single 500 µg MAb dose (DENV1-E99, DENV1-E103, DENV1-E105, or DENV1-E106) was transferred passively by an IP route. Notably, 500 µg of DENV1-E99 or DENV1-E103 provided no protection with animals dying at a rate and time similar to untreated mice (P>0.2). In contrast, DENV1-E105 and DENV1-E106 protected 75 and 82% (P≤0.0001) of mice, respectively, when administered two days after infection. When a single dose of DENV1-E105 or DENV1-E106 MAb was given four days after infection, 20 and 40% (P≤0.03) of mice, respectively were protected from lethal infection (**Fig 7A and B**).

**Figure 8:**
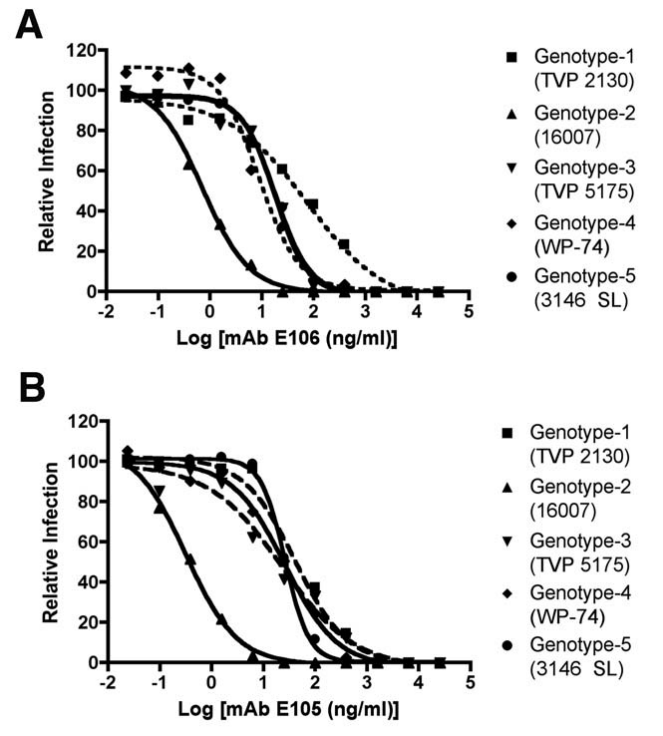
PRNT50 analysis of different DENV-1 genotypes by DENV1-E105 and DENV1-E106. Increasing concentrations of purified (**A**) DENV1-E106 and (**B**) DENV1-E105 were mixed with 10^2^ PFU of DENV-1 strains corresponding to all five genotypes and neutralization was assessed by a standard PRNT assay in BHK or Vero cells (depending on the strain). PRNT50 values were calculated after regression analysis using statistical software. The graph is representatives of at least two independent experiments performed in duplicate.

Given the potency of DENV1-E105 and DENV1-E106 in protection against the genotype 4 West Pac-74 strain, we performed PRNT50 analysis with strains corresponding to the remaining genotypes to assess potential clinical utility of these MAbs. Both DENV1-E105 and DENV1-E106 strongly neutralized infection of all DENV-1 genotypes (**Fig 8A and B**), with PRNT50 values ranging from 0.5 to 59.2 ng/ml. Thus, DENV1-E105 and DENV1-E106 protected AG129 immunocompromised mice as a pre and post-exposure treatment from infection by a heterologous DENV-1 genotype and efficiently inhibited all DENV-1 genotypes in cell culture.

## Discussion

One primary goal of this study was to generate a collection of strongly neutralizing MAbs that would recognize virtually all DENV-1 strains and protect against infection as post-exposure therapy. In an attempt to achieve this, we generated a panel of 79 new MAbs against DENV-1 E protein. Although neutralizing antibodies can be generated against all three domains of the flavivirus E protein [29],[32],[42],[46],[52],[55],[57],[58], we mapped the epitopes of the MAbs with the greatest inhibitory activity primarily to epitopes on DIII, analogous to that observed with MAbs against DENV-2 [25]–[27],[41]. One surprising finding that has not been reported previously was the disparate neutralizing activity and protective potential of individual MAbs against different DENV-1 genotypes. In comparative studies with strains from genotype 2 and genotype 4, virtually all MAbs showed reduced (from 6 to 9,600-fold) PRNT50 values against the heterologous genotype. Because of this, few (5 of 15) MAbs protected as pre-exposure prophylaxis against infection by the heterologous DENV-1 genotype in AG129 mice, and only two (DENV1-E105 and DENV1-E106) showed utility as post-exposure therapy.

Although many DENV strains can cause a similar debilitating febrile illness in humans, there is significant genetic variation (25 to 40% amino acid difference for serotypes and up to 3% variation among genotypes within a serotype) and phenotypic difference in virulence among individual strains of specific serotypes and genotypes [59]–[63]. Previous studies identified the lateral ridge and A-strand epitopes in DIII as targets of strongly neutralizing type-specific and sub-complex-specific neutralizing MAbs against DENV-2 and DENV-3 [26], [27], [41], [64]–[67] and the fusion loop in DII as a recognition site for many cross-reactive neutralizing DENV MAbs [58],[68],[69]. In comparison, the serotype-specific 5H2 neutralizing MAb against DENV-4 localized to the loop between the G and H β-strand in DI [42]. Prior to our study, little was known as to how neutralizing antibodies recognized DENV-1. Although neutralizing anti-DENV-1 MAbs have been generated [44],[45], few were localized to specific amino acids or structural domains. Two neutralizing IgM against DENV-1 were mapped by neutralization escape selection to amino acids E279 and E293, at the hinges between DI and DII or DI and DIII, respectively [46]. While our experiments establish that highly neutralizing antibodies recognize determinants in DIII of DENV-1, we do not rule out the existence of other epitopes in distinct regions. Rather, the panel of MAbs that we obtained may be skewed by the immunization protocol (boosting with recombinant DIII), primary screen, and genetic background of the animals, which influences the B cell repertoire. Indeed, for WNV we have recently identified strongly neutralizing and fusion-blocking human MAbs that poorly recognize recombinant forms of the E protein and map to the DI-DII hinge and dimer contact regions [48].

This is the first study that describes a post-exposure therapeutic effect of a MAb against any DENV strain in any animal model. In particular, DENV1-E105 and DENV1-E106, which neutralized all five DENV-1 genotypes showed efficacy when administered two or four days after infection of highly immunocompromised AG129 mice. In contrast, three other neutralizing MAbs (DENV1-E99, DENV1-E100, and DENV1-103) failed to protect when added a few days after infection even though they functioned as pre-exposure prophylaxis. Although prior studies demonstrated that anti-E MAbs protect against DENV infection in rodents and non-human primates [42],[55],[56],[70],[71], none have reported efficacy when added after infection. It is noteworthy that the two MAbs with the most neutralizing PRNT50 values were the ones with greatest therapeutic efficacy, suggesting, at least for strongly neutralizing MAbs of a given class, a correlation between in vitro and in vivo activity. It is unclear why therapeutic anti-DENV MAbs have not been previously described. Several post-exposure therapeutic MAbs or polyclonal antibody preparations against WNV, JEV, YFV, and tick-borne encephalitis virus have been reported [29], [30], [53], [57], [72]–[77], with some entering human clinical trials [43]. One speculation is that because viremia associated with DENV infection is several log greater than WNV [78]–[80], a condition of viral antigen excess occurs more rapidly in circulation leading to fewer antibody molecules binding to a given DENV virion. As neutralization is determined in part by the stoichiometry of antibody binding [81],[82], only MAbs that can block infection at low fractional occupancy may be capable of neutralizing in vivo.

MAb-based therapeutics against DENV could be complicated by antibody-dependent enhancement of infection (ADE). Sub-neutralizing concentrations of antibody augment infection of Fc-γ receptor-expressing cells possibly by enhancing the efficiency of virus attachment or entry [83],[84]. Thus, the administration of virus-specific MAbs could adversely impact the outcome of DENV infection. In non-human primates, passive transfer of sub-neutralizing concentrations of monoclonal or polyclonal antibody increased viremia although no change in disease status was observed [85],[86]. Based on the possibility of ADE, MAb therapeutics against DENV in humans would appear to have considerable regulatory hurdles. However, recent studies with amino acid substitutions or deletions in the Fc region of recombinant anti-flavivirus antibodies prevented ADE in vitro and in vivo [86]–[88]. As such, studies are underway to engineer mutations into the Fc region of a humanized version of DENV1-E106 that abolishes the possibility of enhancement by abrogating binding to Fc-γ receptors.

Amino acid contact residues of neutralizing MAbs that react with DENV-2 or DENV-3 have been defined by analyzing neutralization escape mutants [40], chimeric DENV variants [89], site-specific DENV-2 mutants [68],[90], peptide mapping [65],[91],[92], yeast surface display of variant E proteins [25],[26],[66], mutant recombinant proteins [27],[41],[67], and by co-crystallography of recombinant DENV proteins and Fab fragments of neutralizing MAbs [25]. Here, we used genetic strategies to generate mutant DIII of DENV-1 on yeast to map antibody recognition residues. The validity of the yeast surface display mapping for identifying critical contact residues has been confirmed by crystallographic studies that resolved the structural interface between WNV and DENV2 DIII Fab fragments [25],[93] as well as DII-fusion loop specific Fab recognition [94]. For neutralizing type-specific DENV-2 MAbs that mapped to specific amino acid residues in DIII, the majority localized to residues in the BC and FG loops within the lateral ridge [26],[41]. Our yeast mapping experiments confirm that the lateral ridge and A-strand on DIII are also recognized by strongly neutralizing anti-DENV-1 antibodies.

The detailed mapping of neutralizing anti-DENV-1 MAbs identified two additional regions on DIII that comprised protective epitope(s): (a) DENV1-E102 and DENV1-E113, which react with multiple DENV serotypes, showed an almost complete loss-of-binding with mutation of residues (Y299, V300, and M301) in the N-terminal linker region. This result is analogous to that described for neutralizing DENV-2 MAbs where the mutation M301G abolished reactivity of five complex-specific and seven serotype-specific MAbs [27],[41]. The binding of the N-terminal linker by MAbs against WNV has been speculated to contribute to neutralization by inhibiting the significant rotation of DIII during the dimer to trimer transition [93]. (b) DENV1-E95, DENV1-E98, DENV1-E104, DENV1-E108, and DENV1-E112 exhibited loss of binding when residues (S390, W391, and K393) in the G-strand were changed. As the G-strand of the DIII is solvent accessible on the surface of the DENV virion [7], it is structurally feasible that recognition of this region by MAbs will inhibit infection. Indeed, in experiments that assessed the reactivity of a panel of complex-specific neutralizing MAbs against DENV-2 and DENV-3 DIII, mutation of G-strand residues also reduced binding [27],[67]. Additionally, X-ray crystallography studies identified residues K388 and N390 in the G-strand of DENV-2 as contacts for the neutralizing sub-complex-specific MAb 1A1D-2 [25]. These studies suggest that DIII is a complex antigen that is recognized by neutralizing MAbs that localize to one of four overlapping regions: the N-terminal linker, A-strand, lateral ridge loops, and G-strand. What remains uncertain is whether binding to specific determinants on DIII influences the mechanism of antibody neutralization of DENV.

Although our most strongly neutralizing MAbs localized to DIII of DENV-1, these antibodies were derived from immunodeficient mice, which may not necessarily reflect the neutralizing antibody response in humans. The antibody repertoire against DENV-1 or other serotypes in humans remains unknown, as B cell profiling at the epitope level has not been performed. Nonetheless, recent studies suggest that the humoral response against flaviviruses in humans may be directed away from DIII neutralizing epitopes [53],[54],[95]. DIII-specific antibodies comprised only a small fraction of the total antibody in DENV immune sera [96],[97]; whether these antibodies contribute to DENV neutralization in vivo remains controversial. Because highly neutralizing antibodies against DIII of DENV-1, DENV-2, and DENV-3 map to the lateral ridge and A-strand epitopes [26],[27],[41],[67], DIII-based vaccines [98]–[101] that skew the humoral response have the potential to elicit highly protective responses.

One intriguing finding was the disparate neutralizing activity of individual MAbs against different DENV-1 genotypes. Infection with one DENV serotype is believed to confer long-term immunity against strains of the homologous but not heterologous DENV serotypes. Based on this, we assumed it would be straightforward to generate DIII-specific therapeutic MAbs that neutralized all five genotypes within the DENV-1 serotype. Indeed, there are limited amino acid changes in DIII among DENV-1 genotypes with a maximum difference of seven amino acids, with genotype 5 the most diverse although the changes are largely conserved and reside in solvent inaccessible regions. Nonetheless, several MAbs in the panel exhibited markedly depressed neutralizing activity against the heterologous West Pac-74 strain. This was perplexing given that the 16007 and West Pac-74 strains differ in DIII by two amino acids, which were not coincident with our epitope mapping data: a T→S change at residue 339 in the C-strand and an A→V change at residue 345 in the C-C′ loop. Indeed, in comparative binding assays with 16007 and West Pac-74 DIII on yeast, no obvious difference in binding at saturating concentrations of MAb was observed. When studies were repeated with selected MAbs in a virus capture ELISA, one MAb (DENV1-E111) with disparate neutralizing potential showed reduced binding to West Pac-74, but others (DENV1-E90 and DENV1-E113) did not. This was corroborated in surface plasmon resonance studies, which showed an ∼10-fold decrease in the K_D_ of binding of DENV1-E111 for West Pac-74 DIII (K. Austin, M. Diamond, D. Fremont, unpublished results). Although further structural studies are warranted, we propose four hypotheses to explain these results: (a) similar to findings with a recently characterized human anti-WNV MAb [48], mutations that abrogate neutralizing activity do not always reduce measurable antibody binding to the virion; (b) because of sequence variation outside of DIII, the E protein of different DENV-1 genotypes may pack differently on the virion. Some components of an epitope for a given MAb may be differentially exposed on virions of distinct genotype; (c) individual loss-of-function mutations identified by yeast display do not necessarily represent the complete footprint on DIII of bound antibody, as mutation of other residues may have allosteric effects; or (d) neutralizing anti-DENV-1 MAbs in our panel may have additional amino acid contacts outside of DIII that vary among the DENV-1 genotypes. Notably, we did not observe detectable binding of any the DIII-specific neutralizing MAbs to a DI-DII protein displayed on yeast (data not shown). Structural studies with Fab-E protein or Fab-virion complexes will be required to address these possibilities.

Regardless of the mechanism, our results suggest that antibodies against one DENV genotype may have decreased inhibitory potency against a heterologous genotype within the same serotype. This has potential implications for assessing the breadth of the protective efficacy of tetravalent DENV vaccines, which are in advanced clinical trials. In addition to evaluating protection against other serotypes, it may be critical to assess whether antibody responses against the vaccine strain of a given serotype neutralize infection of heterologous genotypes equivalently.

## Materials and Methods

### Cells and viruses

Vero, BHK21-15 and Raji-DC-SIGN-R cells were cultured in Dulbecco's Modified Eagle Medium (DMEM) supplemented with 10% fetal bovine serum (FBS) (Omega Scientific) and antibiotics (penicillin G and streptomycin) at 37°C in a 5% CO_2_ incubator. Strains from all five genotypes of DENV-1 included: TVP-2130 (genotype 1), 16007 (genotype 2), TVP-5175 (genotype 3), Western Pacific-74 (genotype 4) and 3146 Sri Lanka (genotype 5). TVP-2130 and TVP-5175 were obtained from Dr. Robert Tesh and the World Reference Center for Emerging Viruses and Arboviruses (University of Texas Medical Branch, Galveston, TX). Western Pacific-74 (West Pac-74) and the 3146 Sri Lanka strain were obtained from Drs. Stephen Whitehead (NIAID, NIH, Bethesda, MD) and Rebeca Rico-Hesse (Southwest Foundation for Biomedical Research, San Antonio, Texas), respectively. Strains corresponding to other DENV serotypes were also used: 16681 (DENV-2), 16652 (DENV-3), and H241 (DENV-4). All viruses were amplified in C6/36 *Aedes albopictus* cells according to previously described protocols [26].

### Generation, purification, and labeling of anti-DENV-1 MAbs

MAbs were generated essentially as described [29] after performing several independent splenocyte-myeloma fusions. To generate anti-DENV-1 MAbs, IFN-αβR^−/−^ C57BL/6 mice were infected with 10^5^ PFU of DENV-1 strain 16007 (genotype 2) via an intraperitoneal route and re-challenged two weeks later with the same strain. Mice with serum having the highest neutralizing titer (>1/1500) were immunized with purified DIII (50 µg, strain 16007) in PBS as a final intravenous boost. Three days later splenocytes were fused to P3X63Ag8.53 myeloma cells [102]. MAbs were subcloned by limiting dilution, isotyped (Pierce Rapid ELISA Kit), and purified using protein A affinity chromatography (Invitrogen). For library sorting experiments, MAbs were labeled with Alexa Fluor 647 or Alexa Fluor 488, using a MAb labeling kit (Molecular Probes) according to the manufacturer's instructions.

### In vitro neutralization assay

Plaque reduction neutralization titer (PRNT) assays were performed with the indicated DENV-1 strains with MAbs or serum on BHK21-15 cells as described previously [26],[80]. PRNT50 values were determined using non-linear regression analysis (Graph Pad Prism4).

### Cloning and expression of DIII of DENV-1 E protein

A cDNA encoding DENV-1 DIII (strain 16007, residues 293 to 400) of the 16007 strain was amplified from viral RNA by reverse transcriptase and high-fidelity Platinum Taq polymerase chain reaction (PCR) according to the manufacturer's instructions (Invitrogen). The PCR product was cloned into the pET21a bacterial expression plasmid (EMD Biosciences) using flanking NdeI and XhoI restriction sites, sequenced, and then expressed in BL21 Codon Plus (Stratagene) *E. coli*. Inclusion bodies containing insoluble aggregates were denatured in the presence of 6 M guanidine hydrochloride and 20 mM β-mercaptoethanol and refolded in the presence of 400 mM L-arginine, 100 mM Tris-base (pH 8.0), 2 mM EDTA, 0.2 mM phenylmethylsulfonyl fluoride, and 5 and 0.5 mM reduced and oxidized glutathione, respectively. Refolded protein was separated from aggregates on a Superdex 75 or 200, 16/60 size exclusion column using fast-protein liquid chromatography (GE Healthcare).

### X-ray crystallographic structure determination of DV1 DIII

DENV-1 DIII was crystallized by vapor diffusion in hanging drops at 20°C at a concentration of 14 mg/ml. The drop contained equal parts protein and well solution composed of 0.1 M imidazole-HCl at pH 7.5, 0.2 M lithium sulfate, and 30% (w/v) polyethylene glycol 3000. Crystals were cryoprotected after a soak in well solution containing 15% glycerol before rapid cooling in a nitrogen gas stream. Data was collected at ALS beamline 4.2.2 (Lawrence Berkeley Laboratories, Berkeley, CA) at 293° K and at a wavelength of 1.29 Å using a CCD detector. The data were processed and scaled in HKL-3000 [103]. Crystallographic phasing was obtained by molecular replacement using the program Phaser [104] and the corresponding DIII fragment from DV2 (PDB accession 1OAN) as a search model. The resulting atomic model was iteratively built in O [105] and refined in REFMAC [106]. The final model with one DENV1 DIII monomer per asymmetric unit has been refined to 2.25Å resolution and contains 103 amino acids (residues 296 to 399 of DIII) and 53 water molecules. The DENV1 DIII domain is similar in structure to that reported for the post-fusion DENV-1 E ectodomain trimer (3G7T) [11] with a root-mean-square difference of 1.6 Å between Cα atom positions for equivalent core residues 296 through 394 [107]. The coordinates and diffraction data have been deposited in the RCSB with accession number 3IRC.

### Domain mapping by yeast surface display

The DNA fragments encoding amino acid residues 1 to 293 (DI-DII) and 294 to 409 (DIII) of DENV-1 E protein were amplified from the DENV-1 strain 16007 or West Pac-74 by RT-PCR with BamHI and XhoI sites added at the 5′ and 3′ end, respectively. The PCR products were cloned as downstream fusions to Aga2 and Xpress epitope tag genes in the yeast surface display vector pYD1 (Invitrogen), under the control of an upstream GAL1 promoter. These constructs were transformed into *Saccharomyces cerevisiae* strain EBY100 [108],[109] using *S.c.* EasyComp Transformation Kit (Invitrogen) to generate yeast that expressed DENV-1 DI-DII or DIII. Individual yeast colonies were grown to logarithmic phase at 30°C in tryptophan-free yeast media containing 2% glucose. Fusion protein expression was induced on the surface by growing yeast for additional 48 hrs in tryptophan-free media containing 2% galactose at 20°C. Yeast cells were washed with PBS supplemented with BSA (1 mg/ml) and incubated with 50 µl of neat culture supernatant or purified MAbs at a concentration of 25 µg/ml. After a 30 minute incubation on ice, yeast were washed in PBS with BSA and then incubated with a goat anti-mouse IgG secondary antibody conjugated to Alexa Fluor 647 (Molecular Probes). After fixation with 1% paraformaldehyde in PBS, yeast cells were analyzed on a FACSArray flow cytometer (Becton-Dickinson) using FloJo software.

### Yeast library construction and screening

To generate a library of DENV-1 DIII variants on yeast, mutation was accomplished by error-prone PCR, using a GeneMorph II random mutagenesis kit (Stratagene). The library was ligated into the pYD1 vector and transformed into XL2-Blue ultracompetent cells (Stratagene). The colonies were pooled and transformed into yeast cells as described above. In some instances, additional site-specific mutations were engineered into DENV-1 DIII by a reverse genetic approach using the Quick Change II Mutagenesis Kit (Stratagene).

For individual MAbs, the DENV-1 DIII mutant library was screened according to a previously described protocol [29]. To identify yeast that had selectively lost binding to a given MAb, the library was stained with an individual Alexa Fluor 647 conjugated MAb for 30 min on ice. To control for surface expression of appropriately folded DIII of DENV-1, yeast cells were subsequently stained for 30 min on ice with an Alexa Fluor 488-conjugated pool of DIII-specific MAbs (DENV1-E90, DENV1-E95, DENV1-E99, DENV1- DENV1-E111, DENV1-E112, and DENV1-E113), and then processed by flow cytometry, The population that was single MAb negative but pool oligoclonal MAb positive was selected and sorted. After four to five rounds of sorting, yeast were plated and individual colonies were tested for binding to individual MAbs by flow cytometry. For clones that had lost binding to the desired MAb of interest, the plasmid was recovered using a Zymoprep yeast miniprep kit (Zymo Research), transformed into XL1-Blue competent *E. Coli* (Stratagene), purified using a QIAprep spin miniprep kit (Qiagen), and sequenced.

### Immunostaining of DENV-infected cells

For the study of binding of DENV-1 MAbs to different DENV strains, Raji-DC-SIGN-R or C6/36 cells were infected with different DENV strains at a multiplicity of infection (MOI) of 1. Depending on the DENV-1 strain, Raji-DC-SIGN-R cells were harvested at 48 or 96 hours and C6/36 cells were harvested at day 7 after infection. Cells were washed, fixed in PBS with 4% paraformaldehyde, and permeabilized in Hanks' balanced salt solution (HBSS) supplemented with 10 mM HEPES (pH 7.3), 0.1% (w/v) saponin (Sigma), and 0.02% NaN_3_. The cells were then incubated with MAbs for 30 minutes on ice, washed and incubated subsequently with an Alexa Fluor 647-conjugated goat anti-mouse IgG (Molecular Probes). After 30 minutes, cells were washed and fixed in PBS with 1% paraformaldehyde and analyzed by flow cytometry. To test whether DENV-1 MAbs cross-reacted with more distantly related flaviviruses, infection and staining experiments were performed with WNV (strain 3000.0259) and Raji-DC-SIGN-R cells as described previously [52].

### DENV-1 virus capture ELISA

The capture ELISA for DENV-1 was based on a published assay for WNV with modifications [48]. Briefly, anti-DENV-1 MAbs were coated at indicated concentrations on MaxiSorp (Nunc) polystyrene 96-well microtiter plates in a sodium carbonate (pH 9.3) buffer. Plates were washed three times in ELISA wash buffer (PBS with 0.02% Tween 20) and blocked for 1 hour at 37°C with ELISA block buffer (PBS, 2% bovine serum albumin, and 0.02% Tween 20). DENV-1 virions (2.5 to 5.0×10^5^ PFU of strain 16007 or West Pac-74) diluted in DMEM with 10% heat-inactivated FBS were captured on plates coated with anti-DENV-1 MAbs for 2 hours at room temperature. Plates were rinsed five times in wash buffer and then incubated with biotinylated 4G2 (1 µg/ml, respectively diluted in ELISA block buffer), which recognizes the fusion loop peptide in DII, for 1 hour at room temperature. Plates were washed again five times and then sequentially incubated with 2 µg/ml of horseradish peroxidase-conjugated streptavidin (Vector Laboratories) and tetramethylbenzidine substrate (Dako). The reaction was stopped with the addition of 2 N H_2_SO_4_ to the medium, and emission (450 nm) was read using an iMark microplate reader (Bio-Rad).

### Mouse experiments

All mouse studies were approved and performed according to the guidelines of the Washington University School of Medicine Animal Safety Committee. IFN-αβR^−/−^ × γR^−/−^ mice on the 129 Sv background (AG129 mice) were a gift from Dr. Skip Virgin (Washington University School of Medicine) and bred in a pathogen-free barrier facility. In prophylaxis experiments, AG129 mice were administered a single dose of individual MAbs one day before infection. Subsequently, mice were challenged with West Pac-74 (genotype 4, 10^6^ PFU) by an IP route and morbidity and mortality were monitored for 60 days. In post-exposure therapeutic experiments, a single 500 µg dose of MAb dose was administered by IP injection two or four days after infection with 10^6^ PFU of West Pac-74.

### Mapping of mutations onto the DENV-1 DIII crystal structure

Figures were prepared using the atomic coordinates of DENV-1 DIII (RCSB accession number 3IRC) using the programs CCP4MG [110]. The alignment of DENV-1 DIII from different genotypes was created with the program ALSCRIPT [111].

### Statistical analysis

All data were analyzed using Prism software (GraphPad software). For survival analysis, Kaplan-Meier survival curves were analyzed by log-rank test. For neutralization assays an unpaired student's T-test was used to determine significance.

## Supporting Information

Table S1 Profile of DENV-1 MAbs
(0.06 MB DOC)

Table S2 Summary of Data Collection and Refinement
(0.03 MB DOC)

## References

[1] Burke DS, Monath TP, Knipe DM, Howley PM (2001). Flaviviruses.. Fields Virology. Fourth Edition ed.

[2] Rico-Hesse R (1990). Molecular evolution and distribution of dengue viruses type 1 and 2 in nature.. Virology.

[3] Holmes EC, Twiddy SS (2003). The origin, emergence and evolutionary genetics of dengue virus.. Infect Genet Evol.

[4] Halstead SB (1988). Pathogenesis of dengue: challenges to molecular biology.. Science.

[5] Monath TP (1994). Dengue: the risk to developed and developing countries.. Proc Natl Acad Sci U S A.

[6] Chambers TJ, Hahn CS, Galler R, Rice CM (1990). Flavivirus genome organization, expression, and replication.. Annu Rev Microbiol.

[7] Kuhn RJ, Zhang W, Rossmann MG, Pletnev SV, Corver J (2002). Structure of dengue virus: implications for flavivirus organization, maturation, and fusion.. Cell.

[8] Zhang Y, Corver J, Chipman PR, Zhang W, Pletnev SV (2003). Structures of immature flavivirus particles.. Embo J.

[9] Modis Y, Ogata S, Clements D, Harrison SC (2003). A ligand-binding pocket in the dengue virus envelope glycoprotein.. Proc Natl Acad Sci U S A.

[10] Modis Y, Ogata S, Clements D, Harrison SC (2005). Variable surface epitopes in the crystal structure of dengue virus type 3 envelope glycoprotein.. J Virol.

[11] Nayak V, Dessau M, Kucera K, Anthony K, Ledizet M (2009). Crystal structure of dengue virus type 1 envelope protein in the postfusion conformation and its implications for membrane fusion.. J Virol.

[12] Mondotte JA, Lozach PY, Amara A, Gamarnik AV (2007). Essential Role of Dengue Virus Envelope Protein N Glycosylation at Asparagine-67 during Viral Propagation.. J Virol.

[13] Pokidysheva E, Zhang Y, Battisti AJ, Bator-Kelly CM, Chipman PR (2006). Cryo-EM reconstruction of dengue virus in complex with the carbohydrate recognition domain of DC-SIGN.. Cell.

[14] Tassaneetrithep B, Burgess T, Granelli-Piperno A, Trumpfheller C, Finke J (2003). DC-SIGN (CD209) mediates dengue virus infection of human dendritic cells.. J Exp Med.

[15] Navarro-Sanchez E, Altmeyer R, Amara A, Schwartz O, Fieschi F (2003). Dendritic-cell-specific ICAM3-grabbing non-integrin is essential for the productive infection of human dendritic cells by mosquito-cell-derived dengue viruses.. EMBO Rep.

[16] Rey FA, Heinz FX, Mandl C, Kunz C, Harrison SC (1995). The envelope glycoprotein from tick-borne encephalitis virus at 2 Angstrom resolution.. Nature.

[17] Bhardwaj S, Holbrook M, Shope RE, Barrett AD, Watowich SJ (2001). Biophysical characterization and vector-specific antagonist activity of domain III of the tick-borne flavivirus envelope protein.. J Virol.

[18] Volk DE, Beasley DW, Kallick DA, Holbrook MR, Barrett AD (2004). Solution structure and antibody binding studies of the envelope protein domain III from the New York strain of West Nile virus.. J Biol Chem.

[19] Yu S, Wuu A, Basu R, Holbrook MR, Barrett AD (2004). Solution structure and structural dynamics of envelope protein domain III of mosquito- and tick-borne flaviviruses.. Biochemistry.

[20] Li L, Lok SM, Yu IM, Zhang Y, Kuhn RJ (2008). The flavivirus precursor membrane-envelope protein complex: structure and maturation.. Science.

[21] Yu IM, Zhang W, Holdaway HA, Li L, Kostyuchenko VA (2008). Structure of the immature dengue virus at low pH primes proteolytic maturation.. Science.

[22] Pierson TC, Fremont DH, Kuhn RJ, Diamond MS (2008). Structural insights into the mechanisms of antibody-mediated neutralization of flavivirus infection: implications for vaccine development.. Cell Host Microbe.

[23] Roehrig JT, Mathews JH, Trent DW (1983). Identification of epitopes on the E glycoprotein of Saint Louis encephalitis virus using monoclonal antibodies.. Virology.

[24] Roehrig JT, Staudinger LA, Hunt AR, Mathews JH, Blair CD (2001). Antibody prophylaxis and therapy for flaviviral encephalitis infections.. Ann NY Acad Sci.

[25] Lok SM, Kostyuchenko V, Nybakken GE, Holdaway HA, Battisti AJ (2008). Binding of a neutralizing antibody to dengue virus alters the arrangement of surface glycoproteins.. Nat Struct Mol Biol.

[26] Sukupolvi-Petty S, Purtha WE, Austin SK, Oliphant T, Nybakken G (2007). Type- and Sub-Complex-Specific Neutralizing Antibodies Against Domain III of Dengue Virus Type-2 Envelope Protein Recognize Adjacent Epitopes.. J Virol.

[27] Gromowski GD, Barrett ND, Barrett AD (2008). Characterization of dengue virus complex-specific neutralizing epitopes on envelope protein domain III of dengue 2 virus.. J Virol.

[28] Diamond MS, Sitati E, Friend L, Shrestha B, Higgs S (2003). Induced IgM protects against lethal West Nile Virus infection.. J Exp Med.

[29] Oliphant T, Engle M, Nybakken G, Doane C, Johnson S (2005). Development of a humanized monoclonal antibody with therapeutic potential against West Nile virus.. Nature Medicine.

[30] Brandriss MW, Schlesinger JJ, Walsh EE, Briselli M (1986). Lethal 17D yellow fever encephalitis in mice. I. Passive protection by monoclonal antibodies to the envelope proteins of 17D yellow fever and dengue 2 viruses.. J Gen Virol.

[31] Gould EA, Buckley A, Barrett AD, Cammack N (1986). Neutralizing (54K) and non-neutralizing (54K and 48K) monoclonal antibodies against structural and non-structural yellow fever virus proteins confer immunity in mice.. J Gen Virol.

[32] Beasley DW, Barrett AD (2002). Identification of neutralizing epitopes within structural domain III of the West Nile virus envelope protein.. J Virol.

[33] Crill WD, Roehrig JT (2001). Monoclonal antibodies that bind to domain III of dengue virus E glycoprotein are the most efficient blockers of virus adsorption to Vero cells.. J Virol.

[34] Roehrig JT, Bolin RA, Kelly RG (1998). Monoclonal antibody mapping of the envelope glycoprotein of the dengue 2 virus, Jamaica.. Virology.

[35] Sanchez MD, Pierson TC, McAllister D, Hanna SL, Puffer BA (2005). Characterization of neutralizing antibodies to West Nile virus.. Virology.

[36] Cecilia D, Gould EA (1991). Nucleotide changes responsible for loss of neuroinvasiveness in Japanese encephalitis virus neutralization-resistant mutants.. Virology.

[37] Seif SA, Morita K, Matsuo S, Hasebe F, Igarashi A (1995). Finer mapping of neutralizing epitope(s) on the C-terminal of Japanese encephalitis virus E-protein expressed in recombinant Escherichia coli system.. Vaccine.

[38] Wu SC, Lian WC, Hsu LC, Liau MY (1997). Japanese encephalitis virus antigenic variants with characteristic differences in neutralization resistance and mouse virulence.. Virus Res.

[39] Schlesinger JJ, Chapman S, Nestorowicz A, Rice CM, Ginocchio TE (1996). Replication of yellow fever virus in the mouse central nervous system: comparison of neuroadapted and non-neuroadapted virus and partial sequence analysis of the neuroadapted strain.. J Gen Virol.

[40] Lin B, Parrish CR, Murray JM, Wright PJ (1994). Localization of a neutralizing epitope on the envelope protein of dengue virus type 2.. Virology.

[41] Gromowski GD, Barrett AD (2007). Characterization of an antigenic site that contains a dominant, type-specific neutralization determinant on the envelope protein domain III (ED3) of dengue 2 virus.. Virology.

[42] Lai CJ, Goncalvez AP, Men R, Wernly C, Donau O (2007). Epitope determinants of a chimpanzee dengue virus type 4 (DENV-4)-neutralizing antibody and protection against DENV-4 challenge in mice and rhesus monkeys by passively transferred humanized antibody.. J Virol.

[43] Diamond MS (2009). Progress on the development of therapeutics against West Nile virus.. Antiviral Res.

[44] Simantini E, Banerjee K (1995). Epitope mapping of dengue 1 virus E glycoprotein using monoclonal antibodies.. Arch Virol.

[45] Chen YC, Huang HN, Lin CT, Chen YF, King CC (2007). Generation and characterization of monoclonal antibodies against dengue virus type 1 for epitope mapping and serological detection by epitope-based peptide antigens.. Clin Vaccine Immunol.

[46] Beasley DW, Aaskov JG (2001). Epitopes on the dengue 1 virus envelope protein recognized by neutralizing IgM monoclonal antibodies.. Virology.

[47] Goncalvez AP, Escalante AA, Pujol FH, Ludert JE, Tovar D (2002). Diversity and evolution of the envelope gene of dengue virus type 1.. Virology.

[48] Vogt MR, Moesker B, Goudsmit J, Jongeneelen M, Austin SK (2009). Human Monoclonal Antibodies Induced by Natural Infection Against West Nile Virus Neutralize at a Post-Attachment Step.. J Virol.

[49] Morrey JD, Siddharthan V, Olsen AL, Roper GY, Wang H (2006). Humanized monoclonal antibody against West Nile virus E protein administered after neuronal infection protects against lethal encephalitis in hamsters.. J Infect Dis.

[50] Morrey JD, Siddharthan V, Olsen AL, Wang H, Julander JG (2007). Defining limits of humanized neutralizing monoclonal antibody treatment for West Nile virus neurological infection in a hamster model.. Antimicrob Agents Chemother.

[51] Zhang W, Chipman PR, Corver J, Johnson PR, Zhang Y (2003). Visualization of membrane protein domains by cryo-electron microscopy of dengue virus.. Nat Struct Biol.

[52] Oliphant T, Nybakken GE, Engle M, Xu Q, Nelson CA (2006). Antibody recognition and neutralization determinants on domains I and II of West Nile Virus envelope protein.. J Virol.

[53] Gould LH, Sui J, Foellmer H, Oliphant T, Wang T (2005). Protective and therapeutic capacity of human single chain Fv-Fc fusion proteins against West Nile virus.. J Virol.

[54] Throsby M, Geuijen C, Goudsmit J, Bakker AQ, Korimbocus J (2006). Isolation and characterization of human monoclonal antibodies from individuals infected with West Nile Virus.. J Virol.

[55] Sultana H, Foellmer HG, Neelakanta G, Oliphant T, Engle M (2009). Fusion loop peptide of the West Nile virus envelope protein is essential for pathogenesis and is recognized by a therapeutic cross-reactive human monoclonal antibody.. J Immunol.

[56] Johnson AJ, Roehrig JT (1999). New mouse model for dengue virus vaccine testing.. J Virol.

[57] Goncalvez AP, Chien CH, Tubthong K, Gorshkova I, Roll C (2008). Humanized monoclonal antibodies derived from chimpanzee Fabs protect against Japanese encephalitis virus in vitro and in vivo.. J Virol.

[58] Goncalvez AP, Purcell RH, Lai CJ (2004). Epitope determinants of a chimpanzee Fab antibody that efficiently cross-neutralizes dengue type 1 and type 2 viruses map to inside and in close proximity to fusion loop of the dengue type 2 virus envelope glycoprotein.. J Virol.

[59] Leitmeyer KC, Vaughn DW, Watts DM, Salas R, Villalobos I (1999). Dengue virus structural differences that correlate with pathogenesis.. J Virol.

[60] Rico-Hesse R (2007). Dengue virus evolution and virulence models.. Clin Infect Dis.

[61] Rico-Hesse R, Harrison LM, Salas RA, Tovar D, Nisalak A (1997). Origins of dengue type 2 viruses associated with increased pathogenicity in the Americas.. Virology.

[62] Armstrong PM, Rico-Hesse R (2003). Efficiency of dengue serotype 2 virus strains to infect and disseminate in Aedes aegypti.. Am J Trop Med Hyg.

[63] Rico-Hesse R (2003). Microevolution and virulence of dengue viruses.. Adv Virus Res.

[64] Lisova O, Hardy F, Petit V, Bedouelle H (2007). Mapping to completeness and transplantation of a group-specific, discontinuous, neutralizing epitope in the envelope protein of dengue virus.. J Gen Virol.

[65] Thullier P, Demangel C, Bedouelle H, Megret F, Jouan A (2001). Mapping of a dengue virus neutralizing epitope critical for the infectivity of all serotypes: insight into the neutralization mechanism.. J Gen Virol.

[66] Rajamanonmani R, Nkenfou C, Clancy P, Yau YH, Shochat SG (2009). On a mouse monoclonal antibody that neutralizes all four dengue virus serotypes.. J Gen Virol.

[67] Matsui K, Gromowski GD, Li L, Schuh AJ, Lee JC (2009). Characterization of dengue complex-reactive epitopes on dengue 3 virus envelope protein domain III.. Virology.

[68] Crill WD, Chang GJ (2004). Localization and characterization of flavivirus envelope glycoprotein cross-reactive epitopes.. J Virol.

[69] Crill WD, Trainor NB, Chang GJ (2007). A detailed mutagenesis study of flavivirus cross-reactive epitopes using West Nile virus-like particles.. J Gen Virol.

[70] Chen Z, Liu LM, Gao N, Xu XF, Zhang JL (2009). Passive protection assay of monoclonal antibodies against dengue virus in suckling mice.. Curr Microbiol.

[71] Kaufman BM, Summers PL, Dubois DR, Eckels KH (1987). Monoclonal antibodies against dengue 2 virus E-glycoprotein protect mice against lethal dengue infection.. Am J Trop Med Hyg.

[72] Engle M, Diamond MS (2003). Antibody prophylaxis and therapy against West Nile Virus infection in wild type and immunodeficient mice.. J Virol.

[73] Ben-Nathan D, Gershoni-Yahalom O, Samina I, Khinich Y, Nur I (2009). Using high titer West Nile intravenous immunoglobulin from selected Israeli donors for treatment of West Nile virus infection.. BMC Infect Dis.

[74] Ben-Nathan D, Lustig S, Tam G, Robinzon S, Segal S (2003). Prophylactic and therapeutic efficacy of human intravenous immunoglobulin in treating west nile virus infection in mice.. J Infect Dis.

[75] Zhang MJ, Wang MJ, Jiang SZ, Ma WY (1989). Passive protection of mice, goats, and monkeys against Japanese encephalitis with monoclonal antibodies.. J Med Virol.

[76] Kimura-Kuroda J, Yasui K (1988). Protection of mice against Japanese encephalitis virus by passive administration with monoclonal antibodies.. J Immunol.

[77] Kreil TR, Eibl MM (1997). Pre- and postexposure protection by passive immunoglobulin but no enhancement of infection with a flavivirus in a mouse model.. J Virol.

[78] Vaughn DW, Green S, Kalayanarooj S, Innis BL, Nimmannitya S (2000). Dengue Viremia Titer, Antibody Response Pattern, and Virus Serotype Correlate with Disease Severity.. J Infect Dis.

[79] Xiao SY, Guzman H, Zhang H, Travassos da Rosa AP, Tesh RB (2001). West Nile virus infection in the golden hamster (Mesocricetus auratus): a model for West Nile encephalitis.. Emerg Infect Dis.

[80] Diamond MS, Shrestha B, Marri A, Mahan D, Engle M (2003). B cells and antibody play critical roles in the immediate defense of disseminated infection by West Nile encephalitis virus.. J Virol.

[81] Pierson TC, Xu Q, Nelson S, Oliphant T, Nybakken GE (2007). The stoichiometry of antibody-mediated neutralization and enhancement of West Nile virus infection.. Cell Host and Microbe.

[82] Klasse PJ (2007). Modeling how many envelope glycoprotein trimers per virion participate in human immunodeficiency virus infectivity and its neutralization by antibody.. Virology.

[83] Gollins S, Porterfield J (1984). Flavivirus infection enhancement in macrophages: radioactive and biological studies on the effect of antibody and viral fate.. J Gen Virol.

[84] Halstead SB (1994). Antibody-dependent enhancement of infection: a mechanism for indirect virus entry into cells. Cellular Receptors for Animal Viruses.

[85] Halstead SB (1979). In vivo enhancement of dengue virus infection in rhesus monkeys by passively transferred antibody.. J Infect Dis.

[86] Goncalvez AP, Engle RE, St Claire M, Purcell RH, Lai CJ (2007). Monoclonal antibody-mediated enhancement of dengue virus infection in vitro and in vivo and strategies for prevention.. Proc Natl Acad Sci U S A.

[87] Mehlhop E, Ansarah-Sobrinho C, Johnson S, Engle M, Fremont DH (2007). Complement protein C1q inhibits antibody-dependent enhancement of flavivirus infection in an IgG subclass-specific manner.. Cell Host and Microbe.

[88] Balsitis SJ, Williams KL, Lachica R, Flores D, Kyle JL (2010). Lethal antibody enhancement of dengue disease in mice is prevented by Fc modification.. PLoS Pathog.

[89] Hiramatsu K, Tadano M, Men R, Lai CJ (1996). Mutational analysis of a neutralization epitope on the dengue type 2 virus (DEN2) envelope protein: monoclonal antibody resistant DEN2/DEN4 chimeras exhibit reduced mouse neurovirulence.. Virology.

[90] Serafin IL, Aaskov JG (2001). Identification of epitopes on the envelope (E) protein of dengue 2 and dengue 3 viruses using monoclonal antibodies.. Arch Virol.

[91] Megret F, Hugnot JP, Falconar A, Gentry MK, Morens DM (1992). Use of recombinant fusion proteins and monoclonal antibodies to define linear and discontinuous antigenic sites on the dengue virus envelope glycoprotein.. Virology.

[92] Trirawatanapong T, Chandran B, Putnak R, Padmanabhan R (1992). Mapping of a region of dengue virus type-2 glycoprotein required for binding by a neutralizing monoclonal antibody.. Gene.

[93] Nybakken G, Oliphant T, Johnson S, Burke S, Diamond MS (2005). Structural basis for neutralization of a therapeutic antibody against West Nile virus.. Nature.

[94] Cherrier MV, Kaufmann B, Nybakken GE, Lok SM, Warren JT (2009). Structural basis for the preferential binding of immature flaviviruses by a fusion-loop specific antibody.. EMBO.

[95] Oliphant T, Nybakken GE, Austin SK, Xu Q, Bramson J (2007). The Induction of Epitope-Specific Neutralizing Antibodies against West Nile virus.. J Virol.

[96] Wahala WM, Kraus AA, Haymore LB, Accavitti-Loper MA, de Silva AM (2009). Dengue virus neutralization by human immune sera: role of envelope protein domain III-reactive antibody.. Virology.

[97] Crill WD, Hughes HR, Delorey MJ, Chang GJ (2009). Humoral immune responses of dengue fever patients using epitope-specific serotype-2 virus-like particle antigens.. PLoS ONE.

[98] Hermida L, Bernardo L, Martin J, Alvarez M, Prado I (2006). A recombinant fusion protein containing the domain III of the dengue-2 envelope protein is immunogenic and protective in nonhuman primates.. Vaccine.

[99] Chu JH, Chiang CC, Ng ML (2007). Immunization of Flavivirus West Nile Recombinant Envelope Domain III Protein Induced Specific Immune Response and Protection against West Nile Virus Infection.. J Immunol.

[100] Chen S, Yu M, Jiang T, Deng Y, Qin C (2007). Induction of tetravalent protective immunity against four dengue serotypes by the tandem domain III of the envelope protein.. DNA Cell Biol.

[101] Valdes I, Bernardo L, Gil L, Pavon A, Lazo L (2009). A novel fusion protein domain III-capsid from dengue-2, in a highly aggregated form, induces a functional immune response and protection in mice.. Virology.

[102] Harlow E, Lane D (1988). Antibodies, A laboratory manual.

[103] Minor W, Cymborowski M, Otwinowski Z, Chruszcz M (2006). HKL-3000: the integration of data reduction and structure solution–from diffraction images to an initial model in minutes.. Acta Crystallogr D Biol Crystallogr.

[104] McCoy AJ, Grosse-Kunstleve RW, Adams PD, Winn MD, Storoni LC (2007). Phaser crystallographic software.. J Appl Crystallogr.

[105] Jones EY, Walker NP, Stuart DI (1991). Methodology employed for the structure determination of tumour necrosis factor, a case of high non-crystallographic symmetry.. Acta Crystallogr A.

[106] Winn MD, Murshudov GN, Papiz MZ (2003). Macromolecular TLS refinement in REFMAC at moderate resolutions.. Methods Enzymol.

[107] Shindyalov IN, Bourne PE (1998). Protein structure alignment by incremental combinatorial extension (CE) of the optimal path.. Protein Eng.

[108] Boder ET, Wittrup KD (1997). Yeast surface display for screening combinatorial polypeptide libraries.. Nat Biotechnol.

[109] Boder ET, Wittrup KD (1998). Optimal screening of surface-displayed polypeptide libraries.. Biotechnol Prog.

[110] Potterton L, McNicholas S, Krissinel E, Gruber J, Cowtan K (2004). Developments in the CCP4 molecular-graphics project.. Acta Crystallogr D Biol Crystallogr.

[111] Barton GJ (1993). ALSCRIPT: a tool to format multiple sequence alignments.. Protein Eng.

[112] Kabsch W, Sander C (1983). Dictionary of protein secondary structure: pattern recognition of hydrogen-bonded and geometrical features.. Biopolymers.

