# Supplementary material for: The Development of Therapeutic Antibodies That Neutralize Homologous and Heterologous Genotypes of Dengue Virus Type 1
Source: PLoS Pathog. 2010 Apr 1;6(4):e1000823. doi: 10.1371/journal.ppat.1000823 (PMC2848552; doi:10.1371/journal.ppat.1000823)
Supplement: Table S1 — Profile of DENV-1 MAbs (0.06 MB DOC) [file ppat.1000823.s001.doc]

**Table S1. Profile of DENV-1 MAbs**

MAb Isotype % Neutralization^a^ DI-II DIII Cross-reactivity

DENV1 E1 IgG1 0 No Yes none

DENV1 E2 IgG1 0 Yes No none

DENV1 E3 IgG3 42 No Yes none

DENV1 E4 IgG2b 0 No Yes DENV-2, 3

DENV1 E5 IgG1 33 No Yes DENV-2, 3

DENV1 E6 IgG1 0 No Yes DENV-2, 3

DENV1 E11 IgG1 42 No Yes none

DENV1 E12 IgG1 33 No Yes DENV-2, 3, 4

DENV1 E13 IgG1 17 No Yes DENV-2, 3

DENV1 E14 IgG1 0 No Yes DENV-2, 3

DENV1 E15 IgG1 0 No Yes DENV-2, 3

DENV1 E16 IgG1 0 No Yes none

DENV1 E17 IgG1 58 Yes No ND

DENV1 E18 IgG2a 58 Yes No ND

DENV1 E19 IgG1 33 Yes No ND

DENV1 E20 IgG1 0 Yes No ND

DENV1 E22 IgG1 0 No Yes none

DENV1 E23 IgG1 0 No Yes none

DENV1 E24 IgG1 50 No Yes none

DENV1 E27 IgG1 17 No Yes DENV-3

DENV1 E29 IgG1 0 No Yes none

DENV1 E30 IgG1 42 No Yes DENV-2, 3

DENV1 E31 IgG1 73 No Yes none

DENV1 E32 IgG1 67 No Yes none

DENV1 E33 IgG1 33 No No none

DENV1 E34 IgG1 25 No Yes none

DENV1 E37 IgG1 0 No Yes none

DENV1 E39 IgG1 38 Yes No none

DENV1 E40 IgM 44 No Yes DENV-2, 3

DENV1 E42 IgG1 11 No Yes DENV-3, 2 (weak)

DENV1 E43 IgG1 33 Yes No none

DENV1 E44 IgG1 0 Yes No none

DENV1 E45 IgG1 14 Yes No DENV-2, 4

DENV1 E47 IgG1 22 Yes No DENV-2, 4

DENV1 E48 IgG1 18 No No none

DENV1 E49 IgG1 38 No Yes none

DENV1 E50 IgM **99** No Yes DENV-2, DENV-3

DENV1 E51 IgG1 10 No Yes none

DENV1 E52 IgG1 22 No Yes DENV-2, 3, 4

DENV1 E54 IgG1 17 No Yes none

DENV1 E55 IgG1 54 No Yes none

DENV1 E56 IgG1 35 No Yes none

DENV1 E57 IgG1 18 No No none

DENV1 E58 IgG1 16 No Yes none

DENV1 E59 IgG2b 12 No Yes DENV-3

DENV1 E62 IgM 36 ND ND none

DENV1 E63 IgG1 48 No Yes DENV-3, 4, 2 (weak)

DENV1 E64 IgG1 33 ND ND none

DENV1 E65 IgG1 25 Yes No none

DENV1 E66 ND 33 ND ND none

DENV1 E67 ND 0 ND ND none

DENV1 E76 IgG2a 32 No No DENV-3

DENV1 E79 ND 0 Yes No DENV-4 (weak)

DENV1 E80 IgG2a 19 No Yes DENV-3

DENV1 E82 ND 14 Yes No DENV-4 (weak)

DENV1 E86 IgG2b 0 No No none

DENV1 E87 IgG2c 0 No Yes DENV-2, 3, 4,WNV

DENV1 E88 IgM 0 Yes No DENV-2, 3

DENV1 E89 IgM 20 No Yes DENV-2, 3

DENV1 E90 IgG2b **100** No Yes DENV-4

DENV1 E91 IgG2b 30 No Yes ND

DENV1 E95 IgG2c **100** No Yes none

DENV1 E97 IgG2c 20 No Yes DENV-2, 3, 4, WNV

DENV1 E98 IgG2c **100** No Yes DENV-2, 3

DENV1 E99 IgG2c **100** No Yes DENV-2, 3

DENV1 E100 IgG3 **100** No No none

DENV1 E101 IgG2c **100** No Yes none

DENV1 E102 IgG2c **100** No Yes DENV-2, 3, 4

DENV1 E103 IgG2c **100** No Yes none

DENV1 E104 IgG2c **96** No Yes none

DENV1 E105 IgG3 **100** No Yes none

DENV1 E106 IgG2c **100** No Yes DENV-4

DENV1 E108 IgG2c **100** No Yes none

DENV1 E109 IgM 30 No Yes none

DENV1 E110 IgG2c 34 No No DENV-2, 3, 4, WNV

DENV1 E111 IgG2c **100** No Yes none

DENV1 E112 IgG2c **100** No Yes none

DENV1 E113 IgG2c **100** No Yes DENV-2, 4

DENV1 E115 IgG2c 30 No No DENV-2, 3, 4

^a^ Neutralizing activity was determined by single endpoint plaque reduction assay on BHK21 or Vero cells with neat hybridoma supernatant cells and 10^2^ PFU of DENV-1 (16007 strain). The data was derived from three independent experiments. ND, indicates not determined.
