# Supplementary material for: The Development of Therapeutic Antibodies That Neutralize Homologous and Heterologous Genotypes of Dengue Virus Type 1
Source: PLoS Pathog. 2010 Apr 1;6(4):e1000823. doi: 10.1371/journal.ppat.1000823 (PMC2848552; doi:10.1371/journal.ppat.1000823)
Supplement: Table S2 — Summary of Data Collection and Refinement (0.03 MB DOC) [file ppat.1000823.s002.doc]

**Table S2. Summary of Data Collection and Refinement**

**Data Collection for DENV-1 DIII (strain 16007)**

Space Group P 2_1_3

Unit Cell (Å) a=88.09, b=88.09, c=88.09

α=90.00, β-=90.00, γ=90.00

Matthews coefficient 5.10

Solvent content (%) 75.88

Completeness (%) 99.1 (98.5)

Rsym(%) 3.9 (69.4)

I/σ 16.9 (1.9)

**Refinement**

Resolution (Å) (outer shell) 35.97-2.25 (2.29-2.25)

R_all_ (%) 21.2

R_work_ overall (outer shell) (%) 21.1 (33.2)

R_free_ overall (outer shell) (%) 21.1 (38.8)

Reflections R_work_/R_free_ 10458/766

RMSD bond length (Å) 0.017

RMSD bond angle (^o^) 1.81

Ramachandran plot

Most Favored/Additional (%) 95.1/99.0

Disallowed (%) 0.97

Average B-values 32.82

Est. Coordinate Error (Å) 0.165
